# Variation in 100 relevant pharmacogenes among emiratis with insights from understudied populations

**DOI:** 10.1038/s41598-020-78231-3

**Published:** 2020-12-04

**Authors:** Zeina N. Al-Mahayri, George P. Patrinos, Sukanya Wattanapokayakit, Nareenart Iemwimangsa, Koya Fukunaga, Taisei Mushiroda, Wasun Chantratita, Bassam R. Ali

**Affiliations:** 1 Department of Pathology, College of Medicine and Health Sciences, United Arab Emirates University, P.O. Box 17666, Al-Ain, United Arab Emirates; 2 Department of Pharmacy, School of Health Sciences, University of Patras, University Campus, Rion, Patras, Greece; 3 Zayed Center for Health Sciences, United Arab Emirates University, Al-Ain, United Arab Emirates; 4 Division of Genomic Medicine and Innovation Support, Department of Medical Sciences, Ministry of Public Health, Nonthaburi, Thailand; 5 Center for Medical Genomics, Faculty of Medicine Ramathibodi Hospital, Mahidol University, Bangkok, Thailand; 6 Laboratory for Pharmacogenomics, RIKEN Center for Integrative Medical Sciences, Yokohama, Japan; 7 Department of Genetics and Genomics, College of Medicine and Heath Sciences, United Arab Emirates University, Al-Ain, United Arab Emirates

**Keywords:** Genetics, Health care

## Abstract

Genetic variations have an established impact on the pharmacological response. Investigating this variation resulted in a compilation of variants in “pharmacogenes”. The emergence of next-generation sequencing facilitated large-scale pharmacogenomic studies and exhibited the extensive variability of pharmacogenes. Some rare and population-specific variants proved to be actionable, suggesting the significance of population pharmacogenomic research. A profound gap exists in the knowledge of pharmacogenomic variants enriched in some populations, including the United Arab Emirates (UAE). The current study aims to explore the landscape of variations in relevant pharmacogenes among healthy Emiratis. Through the resequencing of 100 pharmacogenes for 100 healthy Emiratis, we identified 1243 variants, of which 63% are rare (minor allele frequency ≤ 0.01), and 30% were unique. Filtering the variants according to Pharmacogenomics Knowledge Base (PharmGKB) annotations identified 27 diplotypes and 26 variants with an evident clinical relevance. Comparison with global data illustrated a significant deviation of allele frequencies in the UAE population. Understudied populations display a distinct allelic architecture and various rare and unique variants. We underscored pharmacogenes with the highest variation frequencies and provided investigators with a list of candidate genes for future studies. Population pharmacogenomic studies are imperative during the pursuit of global pharmacogenomics implementation.

## Introduction

Interindividual variations in drug response and side effects are significant challenges in clinical pharmacology. The notion that genetic factors contribute to drug response was further strengthened and consolidated with molecular research. During the last decades, numerous studies aimed to identify the genetic basis of drug response have been conducted. As a result, a plethora of genetic variants-drug pairs were discovered, some of which found their way into drug labels and clinical implementation. Genes known to affect drugs pharmacokinetics (PK) or pharmacodynamics (PD) are recognized as “pharmacogenes”^1–3^. Some pharmacogenes which are known to be significantly involved in multiple drugs pathways or to contribute in severe drug responses have been conventionally called “very important pharmacogenes” (VIPs)^4^. Variations in the form of single nucleotide polymorphisms (SNPs) or copy number variations (CNVs) in genes encoding for drug metabolizing enzymes, drug receptors, drug binding proteins, and drug transporters are key targets of pharmacogenomic (PGx) studies^1–3^.

Technologies in PGx research have evolved from small studies exploring unexplained drug response variability to candidate genes studies to large case–control studies using genome-wide association (GWAS)^5^. High throughput sequencing, namely next-generation sequencing (NGS), facilitated the identification of genetic variation at an exceptional pace, and surpassed all the previously used technologies by discovering rare and common genetic variants at a fast pace and accurate scale^6^. The field of PGx was no exception in leveraging the advantages that NGS offered to genomics and genomic medicine. This was exhibited by the considerable number of studies that utilized NGS data repositories, like the 1000 genome project (1000 GP), and the Exome Aggregation Consortium (ExAC) dataset, to extract the common and rare variants in genes identified in earlier association studies^7–9^.

Such large-scale PGx studies suggested that pharmacogenes are extensively variable^8,10^. Significant differences in the PGx alleles architecture exist between different populations, even if the compared populations were geographically close^11^. Moreover, rare variants are abundant in pharmacogenes, a significant proportion of which are found to have significant effect on drug response. Vary rare variants with allele frequency < 0.01 are not observed usually in clinical trials; however, many of them were found actionable^7^. These rare variants tend to cluster geographically or become private to one population, which elicited the need to develop local catalogs of rare variants across the world^12^.

Population-based studies have the capacity of predicting the occurrence rates of adverse events in communities, which can guide PGx clinical implementation^13^. Targeted NGS capture panels have been a frequent and favorable choice for this kind of research. The lower costs, high speed, and the ability to detect the rare and private variants are all among the reasons that promoted targeted NGS panels for PGx testing at populations scale^14,15^.

The Southeast Asian Pharmacogenomics Research Network (SEAPharm) consortium is a collaborative effort towards achieving sustainable PGx research in the Southeast Asian populations. The consortium’s primary stimulus is to cover the existing knowledge gap in understudied populations^16^. The latest project of SEAPharm aims at resequencing of 100 important pharmacogenes using a targeted sequencing panel, PKSeq, in volunteers recruited from four world geographic areas: South East Asia, South Asia, The Middle East, and Southern Europe. The current study represents the data of resequencing of volunteers from the United Arab Emirates (UAE), a Middle Eastern country with minimal PGx data.

UAE, located on south-eastern part of the Arabian Peninsula in West Asia, is inhabited by almost 10 million population from which the majority are expatriates. The indigenous citizens, also known as Emiratis, make-up about 10–12% of the population. Few genetic studies have been conducted in Emiratis. One recent study illustrated that Emiratis represent a relatively heterogeneous population but with a low interpopulation stratification level^17^.

The current research aims to analyze the landscape of variation identified by the PKSeq panel among healthy Emirati individuals. We intended to determine the allele frequencies, the star alleles, novel alleles, and the participants’ diplotypes, focusing on variants and alleles that have proven or probable clinical actionability. The clinical annotations were extracted from the Pharmacogenomics knowledge database (PharmGKB)^18^, which is a comprehensive repertoire for PGx information that classifies variant-drug pairs according to their accumulated clinical significance. Herein, we have focused on variants with high to intermediate level of evidence. Moreover, we aimed to compare the frequencies of actionable PGx biomarkers from the Emirati population with their frequencies in other populations.

## Methods

### Participants

A group of 100 healthy participants who identified themselves and their parents as Emiratis, participated in this study. Volunteers, including 48 males and 52 females, were recruited from Tawam Hospital, Al-Ain city, Emirate of Abu Dhabi, UAE. All the participants signed an informed consent form to participate in this study. This study was conducted according to Helsinki’s declaration after the approval of the “Tawam Human Research Ethics Committee” (THREC#552). DNA was extracted from whole blood samples, which were collected in EDTA tubes.

### Resequencing

Targeted resequencing of the coding regions and splicing sites of 100 pharmacogenes, including 37 transporters, 30 cytochrome P450 (CYP) enzymes, 10 uridine diphosphate UDP-glucuronosyltransferases (UGT), five flavin-containing monooxygenases (FMO), four glutathione S-transferases (GST), four sulfotransferases (SULT), and 9 other genes active in drug metabolism, and one drug target (*VKORC1*), was conducted using the PKSeq panel (Supplementary-S1). Briefly, we amplified the targeted coding regions (159 kbp) using 1,102 gene-specific primers^19^ and added dual barcodes to the PCR products to differentiate each sample^20^. After purification and quantification of the PCR products, the pooled libraries were sequenced using the MiSeq Reagent Kit v2 (Illumina, San Diego, CA, USA) with an output of 2 × 250 bp. Raw fastq files were processed using a standard analysis pipeline. Reads were aligned to GRCh37/hg19 human reference genome assembly using Burrows-Wheeler (BWA) algorithm. The low-quality bases were removed using Trimmomatic, at average quality 20. Variant calling, variant quality score recalibration, and variant filtering were based on GATK toolset^21^. The minimum sequencing depth was chosen at 30X, with over 98% of targets covered.

### Annotation and effect prediction

Variants annotation and effect prediction were accomplished using ANNOVAR^22^, snpEff^23^, and dbNSFP v4.0^24,25^. ANNOVAR and snpEff annotate and provide effect predictions and frequencies of all variants from any type in the sample. While and dbNSFP v4.0 yields annotations and predictions for the non-synonymous single nucleotide variants only. It also compiles the prediction scores from 29 prediction algorithms including: SIFT, Polyphen2, MutationTaster, MutationAssessor, and others. Besides, providing a comparison of minor allele frequencies (MAF) with the frequencies reported from populations in several databases including: 1000 Gp3 (phase 3 of 1000 GP), UK10K, ExAC, and the genome aggregation databases (gnomAD exome and gnomAD genome). Moreover, it facilitates the predictions of SNVs and splice site variants depending on the SPIDEX and splicing site variants database (dbscSNV)^26^ databases, respectively.

Annovar gives an “unknown” annotation of function when the open reading frame of an exon is not correct^27^. In the cases of “unknown” annotations, these variants were tracked manually using other tools and by revising literature to assign the appropriate annotation.

### Haplotypes and star alleles calling

Stargazer_v1.0.8^28^ has been used to phase the genotypes and to call the haplotypes and corresponding star alleles of 41 genes out of the 100 genes that we targeted. Each gene was genotyped individually in the whole sample using the “Genotype” tool provided with the Stargazer package.

Revising the extracted alleles, the predicted phenotypes and clinical annotations: PharmVar^29^ was revised for *CYP-*alleles, while PharmGKB^18^ was employed as the comprehensive resource for all alleles and for confirming haplotypes annotations, phenotype predictions when possible, and for clinical annotations.

### Statistical methods

Data processing and analysis were applied by R^30^ using suitable tests and their compatible packages.

## Results

### Minor alleles frequencies

**Figure 1:**
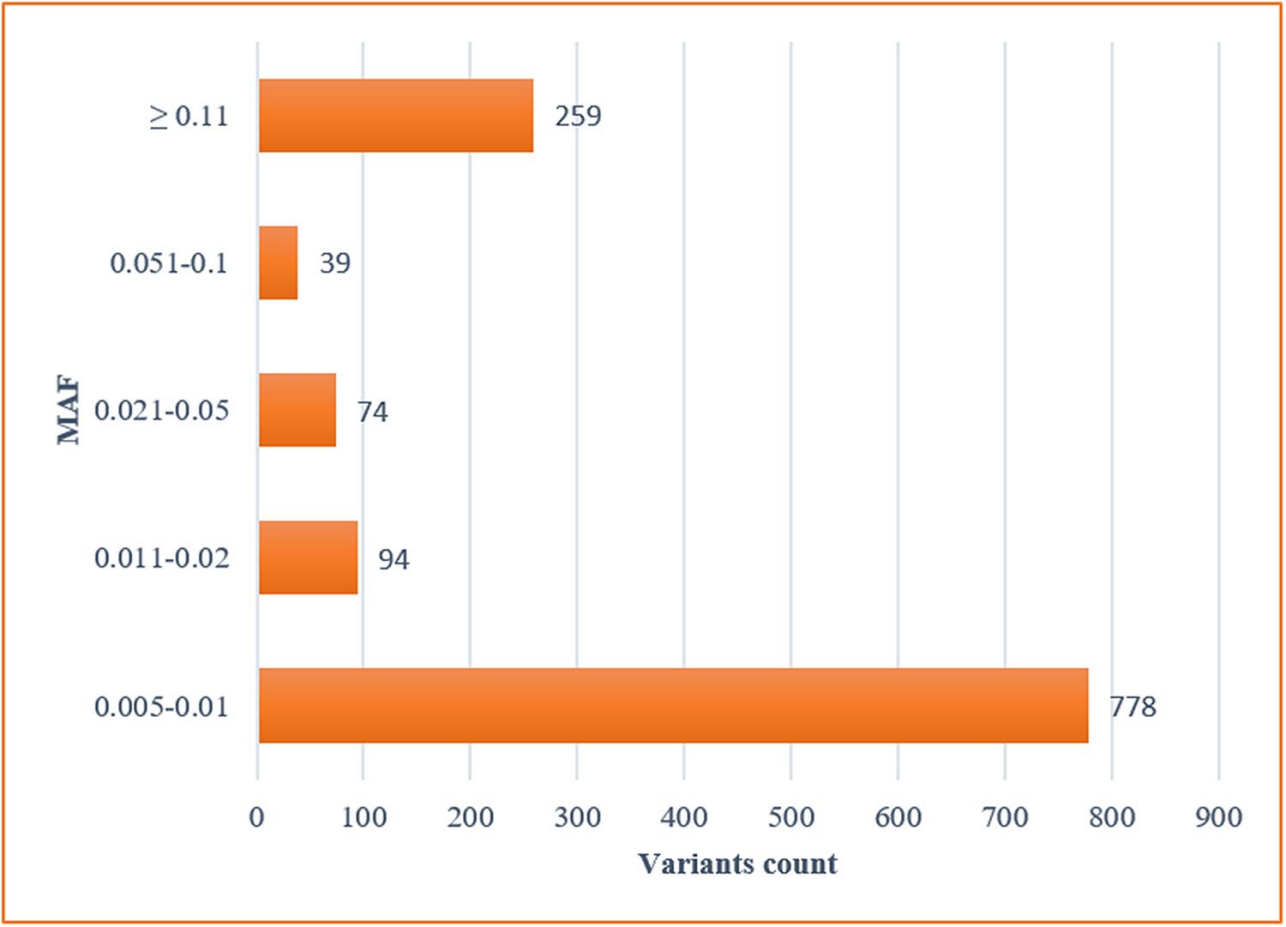
Distribution of variants according to MAF*.* *MAF* minor allele frequency.

Through the targeted sequencing of 100 genes in 100 healthy individuals, 1243 variants passed the quality filters and were considered as high-quality variants. More than half of these (63%) variants were detected in a minor allele frequency (MAF) that is less than or equal to 0.01 (MAF ≤ 0.01), of which the majority (656 variants) were found as singletons; i.e. one minor allele in a heterozygous individual (MAF ≈ 0.005). The distribution of detected variants according to their MAF is illustrated in Fig. 1.

The MAF of variants called by dbNSFP-v4 were compared to other populations from 1000Gp3, UK10K, ExAC, gnomAD-exome and gnomAD-genome databases (sample counts = 1000; 10,000; 60,706; 125,748 and 15,708; respectively). About 30% of the detected variants in our group were unique, i.e., were not found in any of the other compared populations (total other populations count = 213,162).

Comparing the MAF of our variants to their frequencies in the populations included in gnomAD exome and genome databases revealed that 41.7% (n = 537) of the retrieved variants from our population were rare (MAF ≤ 0.01) worldwide and out of these rare variants 154 variants had MAF > 0.01 in our population. In other words, 154 variants are more common in our population than the other compared populations.

### Functional classes of variants and effect predictions

**Figure 2:**
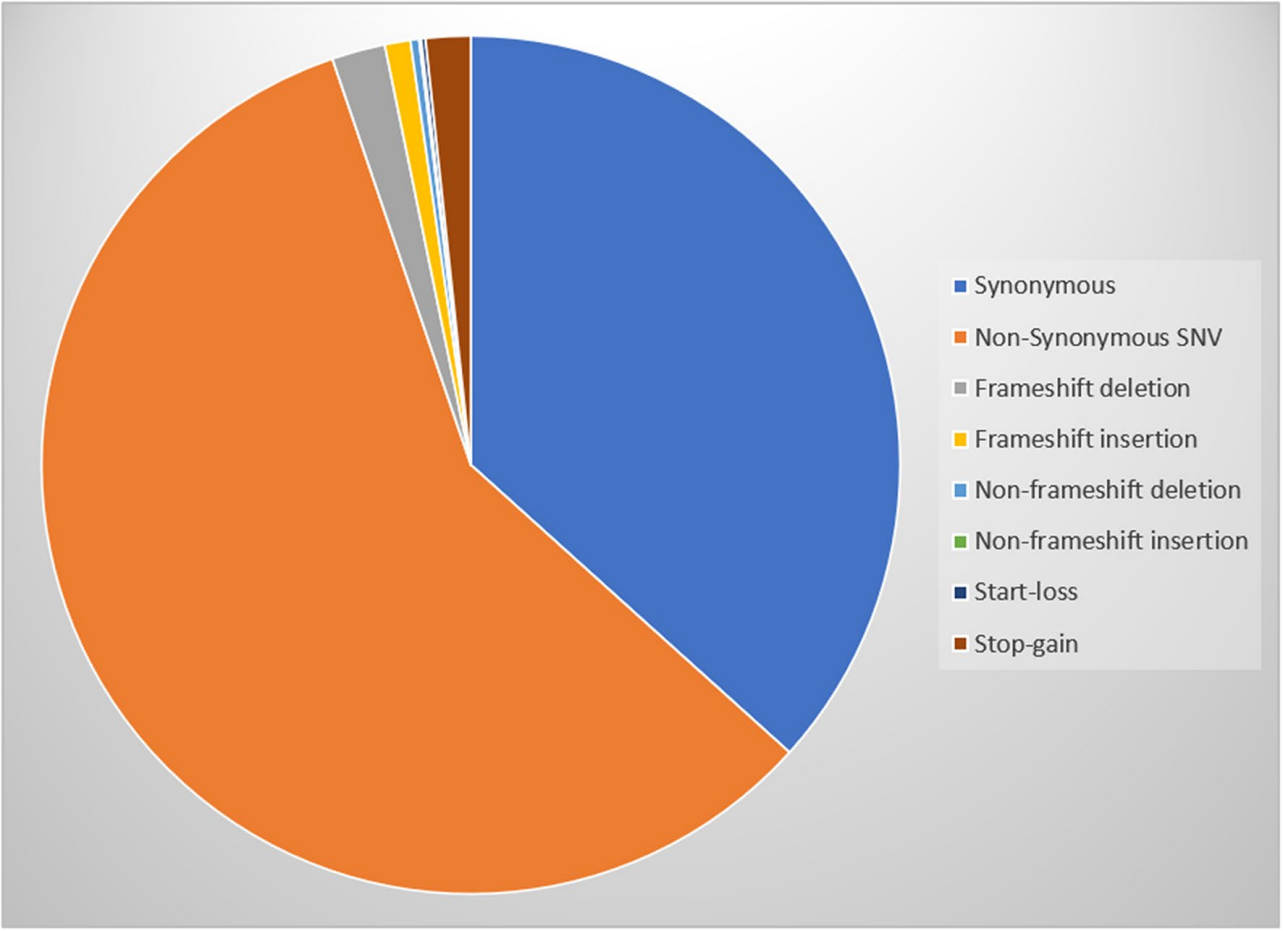
Distribution of the detected variants according to their functional predictions. *SNV* single nucleotide variant.

Most of the detected variants (56.1%) were classified as non-synonymous single nucleotide variants, followed by synonymous variants, followed by the other classes of variants as illustrated in Table 1 and Fig. 2.

**Table 1:** Functional classes of the detected variants.

Variant class	Count	frequency
Synonymous	473	0.37931
Non-synonymous SNV	739	0.592622
Deletion	6	0.004812
Insertion	5	0.00401
Start-loss	2	0.001604
Stop-gain	22	0.017642

Four loss-of-function variants in 4 different genes present in 4 different heterozygous were found. These include rs2020866 in *FMO2*, rs1034422305 in *ABCC3*, G>A at Chr10:104590479 in *CYP17A1* and G>A at Chr19:15770170 in *CYP4F3*. In addition, filtering variants against dbscSNV revealed that 24 of the detected variants were occurring at splice sites.

Contradictory predictions of the variants’ effects were obtained from the different prediction tools used. SIFT predicted that 301 variants are deleterious/damaging variants, which was the highest among all in silico tools, followed by Polyphen with 239 variants, Polyphen2 with 197, FATHM with 131 variants, and MetaLR with 130 variants. MetaLR is a logistic regression-based ensemble prediction that incorporates scores from 10 prediction tools, including the previously mentioned ones and other tools, with allele frequencies in the 1 K genome populations. We have also filtered the variants according to their combined annotation dependent depletion (CADD) scores^31^, in which scores > 20 are considered as likely damaging. The filtration of population specific variants defined 141 variants as the most likely damaging population specific variants (listed in S2_Population specific variants with damaging predictions).

### Haplotypes and star alleles

Stargazer genotyping resulting files were analyzed to extract the frequencies of star alleles, diplotypes, and phenotype prediction. The PharmGKB and PharmVar databases were manually searched to confirm the predictions and to conclude their probable clinical significance.

Some genes presented with more than 15 different diplotypes within our study participants, including *CYP2D6, CYP2B6, DPYD, CYP1B1, CYP4B1* and *NAT2*. Most of the detected diplotypes were predicted to be associated with normal enzymatic activity and were not reported in PharmGKB^27^ to have a clinical significance. However, 27 diplotypes in 11 important genes with a clinical relevance were found among the study participants. These significant diplotypes are listed in Table 2. The full list of star alleles, their associated SNPs, and MAF in our group beside the complete list of diplotypes, their frequencies, and their PharmGKB annotated clinical significance are detailed in supplementary materials “S3_Star alleles and diplotypes”.

**Table 2:** Detected diplotypes with a clinical significance in PharmGKB of a high level of evidence.

Diplotype	Freq	PharmGKB clinical significance (level of evidence)^a,b^
CYP2A6*2/*18	0.05	May have decreased metabolism of nicotine (2A)
CYP2B6*1/*4	0.04	May have an increased metabolism of efavirenz (1A) May have an increased metabolism of bupropion (2A)
CYP2B6*1/*6	0.28	May have a decreased metabolism of efavirenz (1A) May have a decreased metabolism of bupropion (2A)
CYP2B6*1/*9	0.07	
CYP2B6*1/*18	0.01	
CYP2B6*4/*4	0.03	May have an increased metabolism of efavirenz (1A) May have an increased metabolism of bupropion (2A)
CYP2B6*6/*6	0.06	May have a decreased metabolism of efavirenz (1A) May have a decreased metabolism of bupropion (2A)
CYP2B6*9/*9	0.04	
CYP2C8*3/*3	0.02	May have decreased metabolism of ibuprofen (2A)
CYP2C9*1/*2	0.17	May have decreased metabolism and increased plasma concentration of phenytoin, and increased adverse drug reactions (1A), may have increased risk of over-anticoagulation with warfarin and bleeding and patient may require a decreased dose (1A), may have decreased metabolism of piroxicam, meloxicam and tenoxicam (1A), may have altered metabolism and exposure to flurbiprofen (1A), may have decreased metabolism and clearance of ibuprofen (1A), may have an altered likelihood of over-coagulation with acenocoumarol and may require an altered dose (2A)
CYP2C9*1/*3	0.13	
CYP2C9*2/*2	0.02	May have decreased metabolism and increased plasma concentration of phenytoin, and increased adverse drug reactions (1A), may have increased risk of over-anticoagulation with warfarin and bleeding and may require a decreased dose (1A), may have decreased metabolism of meloxicam (1A), may have decreased metabolism and clearance of ibuprofen (1A), may have an altered likelihood of over-coagulation with acenocoumarol and may require an altered dose (2A)
CYP2C9*2/*3	0.01	May have decreased metabolism and increased plasma concentration of phenytoin, and increased adverse drug reactions (1A), may have increased risk of over-anticoagulation with warfarin and bleeding and may require a decreased dose (1A), may have decreased metabolism and clearance of ibuprofen (1A), may have drastically decreased metabolism of tenoxicam (1A), may have an altered likelihood of over-coagulation with acenocoumarol and may require an altered dose (2A)
CYP2D6*1/*10	0.1	May have increased plasma concentration and decreased clearance of paroxetine (1A), May have increased steady state plasma concentration of fluvoxamine (1A)
CYP2D6*2/*10	0.09	May have increased plasma concentration and decreased clearance of paroxetine (1A)
CYP2D6*10/*10	0.02	May have increased plasma concentration and decreased clearance of paroxetine (1A), may have decreased metabolism of tamoxifen to its active metabolite endoxifen, increased likelihood of recurrence and decreased event-free and recurrence-free survival in breast cancer patients (1A) May have decreased metabolism of nortriptyline and increased side effects (1A), May have increased steady state plasma concentration of fluvoxamine and increased GI side effects (1A), May have decreased metabolism or clearance of codeine and decreased response to it (1A), May have decreased clearance of atomoxetine (1A), When treated with amitriptyline may have increased nortriptyline plasma level (1A) May have decreased metabolism of tramadol and should avoid its use (1A), May have decreased metabolism/clearance of venlafaxine and may have decreased tolerance to it (2A), May have increased concentration of tolterodine and its active metabolite (2A), May have decreased metabolism/clearance of metoprolol (2A). May have lower clearance of flecainide (2A), May have decreased metabolism of propafenone and increased side effects (2A) May have reduced metabolism of desipramine (2A), May have decreased metabolism/clearance of risperidone (2A)
CYP4F2*1/*3	0.54	May require a higher dose of warfarin (1A) and acenocoumarol (2A) and phenprocoumon (2A)
CYP4F2*3/*3	0.18	May require a higher dose of warfarin (1A) and acenocoumarol (2A) and phenprocoumon (2A)
NAT2*5/*5	0.19	Patients with two slow acetylator NAT2 alleles (*5/*6/*7): may have decreased metabolism of isoniazid (2A), may have an increased risk of developing isoniazid-induced hepatotoxicity (2A), may have decreased metabolism of hydralazine (2A)
NAT2*6/*6	0.07	
NAT2*6/*7	0.01	
NAT2*5/*6	0.32	
NAT2*5/*7	0.04	
NUDT15*1/*3	0.005	May tolerate lower doses of mercaptopurine (2B)
SLCO1B1*1B/*1B	0.07	May have decreased bioavailability of pravastatin (2A)
UGT1A4*1/*3B	0.21	May have an altered serum concentration of lamotrigine (2B)
UGT1A4*3B/*3B	0.01	May have an increased serum concentration of lamotrigine and improved response (2B)

*Freq* frequency in the current study.

^a^Clinical association with the highest level of evidence (level 1 and 2) are only listed here.

^b^Decrement and increment here is referring to the function of reference alleles (i.e. in comparison to individuals with *1/*1).

### PharmGKB clinical annotations

The detected variants were filtered based on their clinical significance featured in PharmGKB. Ninety-nine SNPs are assigned with at least one clinical annotation with a level of evidence ≥ level 3. Among these, 26 variants were in the four highest levels of evidence 1A, 1B, 2A, and 2B. Table 3 lists these SNPs, the drugs they affect, the kind of this effect as assigned in PharmGKB, besides, their frequencies in our sample.

**Table 3:** Detected SNPs with a high-level significance clinical evidence in PharmGKB with frequencies.

Gene	Variant	Type of interaction	Drug	Level of Evidence	AF
*SLCO1B1*	rs4149056	Toxicity, ADR	Simvastatin	1A	0.187
*NUDT15*	rs116855232	Dosage, Toxicity/ADR	Azathioprine, mercaptopurine	1A	0.005
*DPYD*	rs115232898	Toxicity/ADR	Fluorouracil	1A	0.005
*CYP2C9*	rs1799853	Dosage	Warfarin	1A	0.11
	rs1057910	Dosage	Warfarin	1A	0.07071
*CYP2C19*	rs4244285	Dosage/Efficacy/Toxicity/ADR	Clopidogrel	1A	0.151
		Efficacy/Toxicity/ADR	Citalopram, escitalopram		
		Efficacy/Toxicity/ADR	Amitriptyline		
		Metabolism/Pk	Voriconazole		
*CYP4F2*	rs2108622	Dosage	Warfarin	1A	0.4592
*CYP2B6*	rs3745274	Dosage	Efavirenz	1B	0.31
*ABCB1*	rs1045642	Toxicity/ADR	Methotrexate, nevirapine	2A	0.597
		Efficacy	Ondansetron		
		Dosage	Fentanyl	2A	
		Other	Digoxin		
	rs2032582	Efficacy	Simvastatin, ondansetron	2A	0.611
*ABCC4*	rs1751034	Metabolism/PK	Tenofovir	2A	0.826
*ABCG2*	rs2231142	Efficacy	Rosuvastatin	2A	0.061
		Dosage, Efficacy	Allopurinol		
*CES1*	rs71647871	Efficacy	Clopidogrel	2A	0.02
*CYP2B6*	rs2279343	Metabolism/PK	Efavirenz	2A	
	rs3745274	Other	Nevirapine	2A	0.31
		Toxicity/ADR	Efavirenz		
		Dosage	Methadone		
	rs28399499	Other	Nevirapine	2A	0.0101
		Metabolism/PK	Efavirenz	2A	
		Toxicity/ADR	Nevirapine	2B	
*CYP2C8*	rs10509681	Efficacy, Toxicity/ADR, Metabolism/PK	Rosiglitazone	2A	0.1364
*CYP2C9*	rs7900194	Dosage, Toxicity/ADR	Warfarin	2A	0.02
	rs1057910	Toxicity/ADR	Anti-inflammatory agents, non-steroids, celecoxib, diclofenac	2A	0.07071
		Dosage	Celecoxib	2A	
		Toxicity/ADR	Acenocoumarol, warfarin		
		Dosage, Toxicity/ADR	Acenocoumarol		
*CYP2C19*	rs4244285	Other	Imipramine, clomipramine	2A	0.151
		Metabolism/PK	Citalopram, escitalopram		
		Efficacy /Toxicity/ADR	Aspirin, clopidogrel		
*CYP4F2*	rs2108622	Dosage	Phenprocoumon	2A	0.4592
		Dosage	Acenocoumarol		
*UGT1A1*	rs4148323	Other	SN-38	2A	0.005
		Other	Irinotecan		
*SLCO1B1*	rs4149056	Toxicity/ADR	Cerivastatin	2A	0.187
		Other	Rosuvastatin		
		Metabolism/PK	Simvastatin acid	2B	
*NAT2*	rs1799930	Toxicity/ADR, Metabolism/PK	Ethambutol, isoniazid, pyrazinamide, rifampin	2A	0.315
	rs1041983	Toxicity/ADR	Ethambutol, isoniazid, pyrazinamide, rifampin		0.3333
*GSTP1*	rs1695	Toxicity/ADR	Platinum compounds	2A	0.3163
		Efficacy	Fluorouracil, oxaliplatin		
		Efficacy, Toxicity/ADR	Cyclophosphamide, epirubicin		
		Toxicity/ADR	Cisplatin	2B	
*VKORC1*	rs61742245	Dosage	Warfarin	2A	0.005
*CES1*	rs71647871	Efficacy	Clopidogrel	2B	0.02041
*SLC28A3*	rs7853758	Toxicity/ADR	Anthracyclines and related substances	2B	0.1111
*UGT1A4*	rs2011425	Other	Lamotrigine	2B	0.1173

*ADR* adverse drug reaction, *AF* allele frequency in the current group.

### Comparison with other populations

Chi-square and Fisher Exact tests were used to compare the MAF of the 25 variants of the highest level of clinical evidence between the current studied population and their MAF in gnomAD eight subpopulations: African, South Asia, East Asia, Europe non-Finnish, Europe-Finnish, Ashkenazi Jewish, Latino and Other. The allele counts and numbers were retrieved from gnomAD browser^32^ (last accessed 14 Jun 2020). The comparison resulted in some significant and some non-significant differences between the frequencies at the studied UAE population and the compared populations. The results are listed in Table 4.

**Table 4:** Comparison of MAF at the clinically actionable variants in UAE population and gnomAD populations.

Gene	Rs-number	UAE	MAF in GnomAD populations (p-values from Chi^2^ tests)							
			Af	SA	EA	EnF	EF	AJ	Latin	Other
*ABCB1*	rs1045642	0.6	**0.799 (< 10**^**–5**^**)**	**0.3952 (< 10**^**–5**^**)**	0.6321 (0.3468)	0.4663 (3*10^**–3**^**)**	**0.386 (< 10**^**–5**^**)**	0.6441 (0.197)	0.548 (0.193)	**0.5127 (0.024)**
*ABCB1*	rs2032582	0.6	**0.916** **(< 10**^**–5**^**)**	**0.3494 (< 10**^**–5**^**)**	**0.4765 (0 .001)**	0.547 (0.195)	0.469 (6*10^**–4**^**)**	0.6247 (0.477)	0.548 (0.201)	0.5512 (0.23)
*ABCC4*	rs1751034	0.8	**0.74 (0.007)**	**0.9062 (0.0002)**	0.787 (0.208)	0.817 (0.82)	0.843 (0.571)	**0.743 (0.0107)**	**0.74 (0.009)**	0.7974 (0.362)
*ABCG2*	rs2231142	0.06	**0.027 (0.007)**	0.093 (0.157)	**0.307 (< 10**^**–5**^**)**	0.1036 (0.067)	0.073 (0.630)	0.0653 (0.933)	**0.224 (< 10**^**–5**^**)**	**0.1084 (0.046)**
*CES1*	rs71647871	0.02	**0.002 (0.0008)**	**0.0061 (0.0344)**	**0.0001 (< 10**^**–5**^**)**	0.0146 (0.5408)	0.012 (0.299)	0.026 (0.821)	0.007 (0.052)	0.0156 (0.5528)
*CYP2B6*	rs2279343	0.06	**0.24 (< 10**^**–5**^**)**	**0.2516 (< 10**^**–5**^**)**	0.1547 (4*10^**–4**^**)**	**0.1072 (0.0457)**	0.101 (0.075)	0.1536 (5*10^**–4**^**)**	0.170 (8*10^**–6**^**)**	0.1655 (8*10^**–4**^**)**
*CYP2B6*	rs28399499	0.01	0.07 (1*10^**–4**^**)**	**0.0001 (0.0008)**	0 (NA)	**0.0001 (0.0005)**	0 (NA)	0 (NA)	0.003 (0.156)	**0.0015 (0.0456)**
*CYP2B6*	rs3745274	0.3	0.370 (0.088)	**0.3894 (0.0262)**	0.1908 (3*10^**–5**^**)**	**0.240 (0.025)**	0.1914 (3*10^**–5**^**)**	0.2684 (0.2178)	0.31 (0.96)	0.2607 (0.1378)
*CYP2C19*	rs3758581	0.08	**0.012 (< 10**^**–5**^**)**	0.11 (0.141)	**0.037 (0.009)**	0.0656 (0.6952)	0.0559 (0.3126)	0.0822 (0.8114)	**0.037 (0.007)**	0.0609 (0.5027)
*CYP2C19*	rs4244285	0.15	0.178 (0.388)	**0.324 (< 10**^**–5**^**)**	**0.3075 (< 10**^**–5**^**)**	0.1468 (0.9317)	0.175 (0.4390)	0.132 (0.4883)	**0.101 (0.027)**	0.1595 (0.8418)
*CYP2C8*	rs10509681	0.14	**0.02 (< 10**^**–5**^**)**	**0.0408 (< 10**^**–5**^**)**	**0.0003 (< 10**^**–5**^**)**	0.1134 (0.3656)	0.1107 (0.3023)	0.0992 (0.1081)	0.068 (2*10^**–3**^**)**	0.0993 (0.1115)
*CYP2C9*	rs1057910	0.07	**0.012 (< 10**^**–5**^**)**	0.109 (0.103)	**0.033 (0.007)**	0.068 (0.99)	0.063 (0.747)	0.084 (0.58)	**0.038 (0.029)**	0.063 (0.751)
*CYP2C9*	rs1799853	0.11	**0.022 (< 10**^**–5**^**)**	0.047 (5*10^**–5**^**)**	**0.0004 (< 10**^**–5**^**)**	0.126 (0.561)	0.114 (0.933)	0.135 (0.348)	**0.068 (0.029)**	0.1035 (0.8569)
*CYP2C9*	rs7900194	0.02	**10**^**–5 (**^**< 10**^**–5**^**)**	0 (NA)	0 (NA)	0 (NA)	0 (NA)	0 (NA)	0 (NA)	0 (NA)
*CYP4F2*	rs2108622	0.46	**0.09 (< 10**^**–5**^**)**	0.398 (0.093)	**0.249 (< 10**^**–5**^**)**	**0.287 (< 10**^**–5**^**)**	**0.196 (< 10**^**–5**^**)**	**0.346 (0.001)**	**0.229 (< 10**^**–5**^**)**	**0.29 (< 10**^**–5)**^
*DPYD*	rs115232898	0.01	0.021 (0.137)	3*10^**–6 (**^**0.013)**	**0.00005 (0.02)**	4*10^**–4**^ **(0.01)**	0 (NA)	0 (NA)	0.0008 (0.146)	0.002 (0.299)
*GSTP1*	rs1695	0.316	**0.448 (**3*10^–4^)	0.287 (0.411)	**0.177 (< 10**^**–5**^**)**	0.333 (0.681)	0.27 (0.177)	**0.2143 (0.0008)**	**0.5175 (< 10**^**–5**^**)**	0.3348 (0.6429)
*NAT2*	rs1041983	0.333	**0.451 (0.001)**	**0.428 (0.009)**	0.419 (0.02)	0.314 (0.607)	0.27 (0.07)	0.37 (0.29)	0.304 (0.414)	0.328 (0.940)
*NAT2*	rs1799930	0.315	**0.257 (0.077)**	0.362 (0.1892)	0.258 (0.08)	0.288 (0.45)	0.238 (0.013)	0.355 (0.2784)	0.156 (**< 10**^**–5)**^	0.276 (0.252)
*NUDT15*	rs116855232	0.005	**0.001 (0.512)**	**0.067 (0.0009)**	**0.1048 (< 10**^**–5**^**)**	0.0035 (0.8)	0.023 (0.15)	0.004 (0.772)	0.06 (0.002)	0.021 (0.202)
*SLC28A3*	rs7853758	0.111	**0.311 (< 10**^**–5**^**)**	0.127 (0.59)	0.151 (0.145)	0.137 (0.341)	0.07 (0.02)	0.2132 (0.0006)	0.1995 (0.0025)	0.156 (0.101)
*SLCO1B1*	rs4149056	0.187	**0.03 (< 10**^**–5**^**)**	**0.05 (< 10**^**–5**^**)**	0.13 (0.01)	0.159 (0.327)	0.21 (0.43)	0.179 (0.87)	**0.112 (0.001)**	0.165 (0.471)
*UGT1A1*	rs4148323	0.005	7*10^–4^ (0.14)	0.02 (0.193)	**0.153 (< 10**^**–5**^**)**	0.002 (0.33)	**0.05 (< 10**^**–5**^**)**	0.005 (1)	0.024 (0.098)	0.012 (0.732)
*UGT1A4*	rs2011425	0.117	0.099 (0.482)	**0.1955 (0.008)**	**0.208 (0.002)**	0.088 (0.18)	**0.053 (10**^**–4**^**)**	0.1045 (0.641)	0.1243 (0.8521)	0.1024 (0.5761)

*Af* African, *AJ* Ashkenazi Jewish, *EA* East Asians, *EnF* European non-Finnish, *EF* European Finnish, *SA* South Asia, *UAE* frequencies in the current study, *MAf* minor allele frequency.

*The upper number describes the MAF at the selected variant in the mentioned population, while the lower number describes P value. Cells in bold highlight a significant difference (significant at p < 0.05).

**Figure 3:**
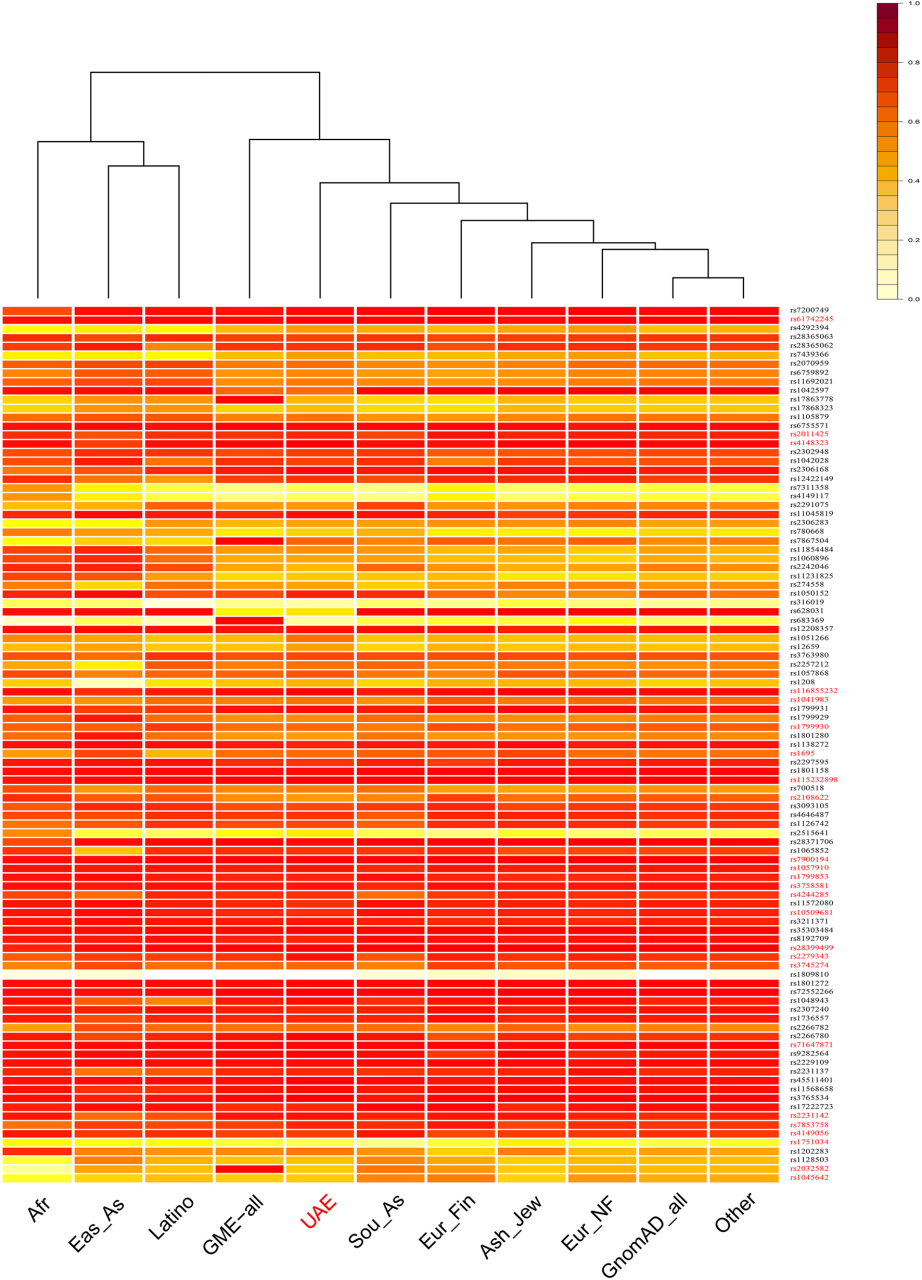
Heatmap for comparison of MAF in UAE to 10 other populations*.* Top right: color scale according to allele frequency, Red rs-numbers designate variants that have level 1 and 2 PhramGKB annotations, while the black rs-numbers designates variants with level-3 PhramGKB annotations. *Afr* African, *Eas_As* East Asians, *GME-all* Greater Middle East whole sample, *UAE* frequencies in the current study, *Sou_As* South Asia, *Eur_Fin* European Finnish, *GnomAD_All* frequencies from GnomAD whole samples.

Furthermore, The MAF of all the retrieved 99 PharmGKB clinically annotated variants, were compared to their MAF in the eight gnomAD subpopulations and with their frequencies in the “Greater Middle East Genome” (GME) project^33^ (last accessed 14 Jun 2020), GME_all, as reported from the total GME samples (n = 2497). The results are depicted in a heatmap with a dendrogram (Fig. 3). The clustering of the compared populations illustrates that our sample (UAE) have clustered close to gnomAD South Asia, and the GME_all populations.

## Discussion

In the current study, we determined the MAF, star alleles, and common diplotypes of important pharmacogenes in a group of healthy Emirati individuals. It is one of the very few studies from the Middle East region that covers drug-related genes at this number and analytical levels with a focus on clinically actionable PGx biomarkers. We have also illustrated, and for the first time, multiple comparisons of a group of biomarkers in relevant pharmacogenes between our population and other populations.

Our results suggested that a significant number of the detected variants were rare, which is in concordance with findings from different population studies of pharmacogenes using NGS^34,35^. A high percentage of these rare variants were novel. Multiple extensive studies demonstrated that rare variants are abundant (one variant every 17 bases) and tend to become private to one population^12^. In contrast to disease-associated variants, the high MAF of a PGx variant is not an indicator of its neutral effect^36^, and vice versa, the rarity of a variant is not a sufficient indicator of its deleterious effect. However, there is excellent value of detecting rare variants and population-specific variants in pharmacogenes as these are not generally included in popular genotyping platforms, which are commonly used in clinical practice^37^.

Beside reporting the individual SNPs, we analyzed the sequencing data by an innovative bioinformatic tool, Stargazer, to define the haplotypes, the star alleles, and diplotypes^28^. The manual confirmation of the retrieved alleles proved that the haplotyping was accurate but with some deficiencies which hampered the full utilization of the tool capabilities. For example, at *CYP2C19*, which is a very important pharmacogene, CYP2C19*17 is determined by rs12248560 which is located on the 5'-flanking area and is not covered here. Accordingly, individuals carrying this increased-function alleles could not be detected. Many clinically approved actionable PGx haplotypes have their key variants in the intronic and gene flanking regions. Hence, covering these variants should be considered during study design for the full utilization of sequencing results. Despite this limitation, a list of star alleles in relevant pharmacogenes has been curated and is made available for future research.

Regarding the functional predictions, we reported an inconsistency between different in silico tools predictions, which is not surprising. Indeed, the interpretation of any novel or population-specific variant should be made through testing in expression systems. However, this is not feasible with the overwhelming number of polymorphisms detected in the NGS era^38^. Thus, computational methods are indispensable but with an understanding of their limitations. In silico prediction tools are built on algorithms utilizing secondary structures of proteins, proteins stability, or the sequence conservation across species. Pharmacogenes are believed to be under lower evolutionary conservation, which makes sequence conservation for variants in pharmacogenes unreliable information for an in silico prediction tool. Moreover, these tools are trained on variants involved in disease, so they are not ideally calibrated for PGx variants^36^. All these factors can explain the contradiction between prediction tools outcomes and the clinical findings of some PGx variants and highlights the importance of functional studies. In a recent comparison of the performance of different in silico algorithms for the effect prediction of pharmacogenomic variants, CADD was amongst the best in terms of performance^38^. Meanwhile, any study concerned with analyzing PGx variations should consider the accumulated evidence curated in public archives and knowledge databases such as PharmGKB. In the following paragraphs, we discuss the retrieved variants and their potential clinical effects according to the PharmGKB, classified according to genes groups.

ATP-binding cassette (ABC) transporter genes contribute to the transportation of endogenous and exogenous molecules, including drugs. Accumulating evidence of the association between variants in *ABC* genes and drugs’ PK and PD resulted in some level-2 clinical annotations included in PharmGKB^39^. In the current Emirati group, we detected the minor alleles at a high frequency at the three most studied SNPs in *ABCB1*, rs1045642, rs2032582, and rs1128503. A haplotype composed of the three SNPs was previously defined and studied in different ethnicities, given the high linkage disequilibrium between these SNPs^40^. In the current group, a haplotype comprising the alternative allele at the three variants was the most common haplotype with a frequency of 0.523. Despite the recurrent evidence of *ABCB1* haplotypes association with simvastatin toxicity, there is not sufficient evidence yet to support its clinical utility^39^. In contrast, the minor alleles at the first two SNPs (rs1045642, rs2032582) are categorized as having level-2 evidence of association with methotrexate, ondansetron, fentanyl, digoxin, and nevirapine. 80% of individuals in our group had one of these alleles. The relatively high frequency of actionable variants in *ABCB1* underscores it as an important candidate for association studies in the Emirati population.

Variants in other genes from this family include rs2231142 in *ABCG2* and rs1751034 in *ABCC4. *Both have level-2 clinical annotations in PharmGKB. The alleles at rs2231142 in *ABCG2* have a probable association with rosuvastatin response and concentration, however, contradictory results were retrieved from different populations^41^. Similarly, moderate evidence supports the association of the same variant with allopurinol and supports the association of rs1751034 in *ABCC4* with the clearance of the anti-HIV drug tenofovir^42^.

Cytochrome p-450 (CYP) enzymes are known to catalyze the oxidation reactions of a broad range of drugs. Fifty-seven genes encode these enzymes, of which 12 of them are responsible for the oxidation of almost 75% of drugs^43^. In the current study, we covered 30 *CYP* genes, including the most essential 12 members. *CYP* family harbored the highest variants count among the studied pharmacogenes in the present study, and *CYP4F12* was the member with the largest number of variants. This gene has been previously described as the *CYP* gene with the highest variation rate^8^. *CYP2D6* was the second most polymorphic gene from this group, from which 11 variants were key markers in clinically actionable haplotypes. However, *CYP2D6* variation tends frequently to be overestimated due to the difficulty of assessing this gene by short-read sequencing^8^.

Seventy-two individuals had missense variant at rs2108622 (or CYP4F2*3), of which 18 of them had two alternative alleles at this SNP. The reported MAF (0.459) is significantly higher than all other gnomAD populations except for southeast Asia (Table 4). Individuals with the minor allele at rs2108622 require higher doses of warfarin to achieve the target international normalized ratio (INR) if they needed warfarin.

While at *CYP2C9*, which is another *CYP* gene known to affect warfarin dose, 30 individuals had one of its low or no function alleles (CYP2C9*2 or *3) and three individuals were homozygous to one of these alleles. Patients using warfarin as an anticoagulant and have CYP2C9*2 or *3 have a higher risk of warfarin-induced bleeding. Indeed, three genes, *CYP4F2, CYP2C9* from warfarin metabolic pathway, and *VKORC1*, encoding warfarin target enzyme, have well-studied genetic biomarkers of warfarin dose. Guidelines have been developed to guide the dosing of warfarin, and some algorithms are now used to adjust the dose according to genetic and clinical factors^44^. However, there is a need to evaluate the dosing algorithms’ implication in different populations as they showed unequal performance for different races^45^.

*CYP2D6* is another crucial member of cyp-450 enzymes that metabolizes 25% of the commonly used drugs^46^. Two decreased function alleles were found in the current group; CYP2D6*10, and CYP2D6*41. These alleles are known to interact with many antidepressants, the estrogen receptor modulator tamoxifen, and to be a contraindication of using tramadol. Individuals carrying two decreased function alleles are vulnerable to many drug interactions. Noteworthy, the currently interpreted *CYP2D6* haplotypes and diplotypes are not conclusive. *CYP2D6* is one of the most challenging genes for interrogation by short-read NGS. This complexity is due to its homology to the pseudogenes *CYP2D7* and *CYP2D8*, and because it is known to display duplications and structural rearrangements that are not detected by conventional techniques^47^. Accordingly, the deficiency in reporting all the possible alleles is not unexpected. We reported here the main alleles found, though further studies are needed on this complex gene.

*CYP2D6* is one of the most studied cytochrome P-450 enzymes with few studies originating from Arab populations. In the UAE, a single previous research has examined the alleles and haplotypes of *CYP2D6 *in 151 UAE nationals^48^. There was no significant difference (p = 0.72) between the reference CYP2D6*1 allele count in our group (37.5%) and the previous UAE group (39.1%). Moreover, and similar to Qumsieh and coworkers’ findings, CYP2D6*41 was a common low function allele reported in both groups.

In contrast to the reported heterogeneity of *CYP2D6*, all participants in the current study were reported to have the ancestral allele of *CYP2A1 *(i.e., CYP1A1*1), and no variants were detected in any individual. These findings are close to one previous study on Emirati volunteers, where CYP1A1*1 allele was present in 90.5% of the participants (1152 alleles)^49^.

*CYP2C19 *encoded enzyme catalyzes the metabolism of wide range of commonly prescribed drugs including clopidogrel, imipramine and phenytoin^50^. In the current group important variants with multiple interactions were detected in *CYP2C19*, including rs4244285. This variant has been reported with high prevalence in the populations from the Middle East^51^. This allele interacts with clopidogrel and with many antidepressants, which some of them are also substrates to *CYP2D6*. The interaction of these two genes and their effect on antidepressants are important pharmacogenomic interactions that warrant further evaluation. Nevertheless the limited study numbers and sizes that have ever evaluated *CYP2C19* alleles in participants rather than Caucasians^50–52^.

Solute carriers (SLC) are considered one of the largest families of membrane proteins acting in transporting a plethora of organic and inorganic substrates. Some of the SLC members play a critical role in drug transport; hence, drug PK and PD^10^. One of the most extensive studies on *SLC* demonstrated that variants of PGx significance in this family are highly differentiated between populations, and 42% of these variants differed in up to more than fivefold between the studied populations (EXAC-populations)^10^.

*SLCO* genes are a family closely related to *SLC,* but it encodes transporters of the organic anion transporting polypeptides (OATPs). rs4149056 in *SLCO1B1* is the most studied SNP in this family of genes. It is known to impair simvastatin hepatic clearance and to increase the risk of induced myopathy^53^. In the current group, rs4149056 was detected with MAF = 0.187, which was significantly higher (p < 0.05) than its frequency in South Asians, East Asians, Latino and African populations. Seven homozygous individuals had another variant in the same gene, rs2306283, which is the critical variant in SLCO1B1*1B. Both *SLCO1B1* variants partially explained the inter-ethnic differences in statin exposure and side effects^53^.

The uridine diphosphate glucuronosyltransferase (UGT) enzymes encoded by *UGT* genes catalyze the glucuronidation of various compounds to facilitate their elimination. variations in *UGT* genes were the subject of a number of pharmacogenetic research^54^. In the current study, two level-2 variants were detected. Rs4148323 in *UGT1A1*, which has been studied more frequently in Asian populations with conflicting evidence about its association with irinotecan-induced toxicities^54^. The other level-2 variant rs2011425 in *UGT1A4* (UGT1A4*3B) known to be associated lamotrigine concentration with inconsistent evidence, and further research is needed^55^.

Human cytosolic sulfotransferase (SULT) enzymes catalyze sulfate conjugation of many compounds playing an essential role in drug metabolism^56^. *SULT* gene family includes 13 members, of which four of them were sequenced in the current study. Only two of the retrieved variants are annotated in the PharmGKB but with evidence level 3. One variant, rs1042028, is of particular importance as it is located in *SULT1A1* which is active in the biotransformation of procarcinogens^56^. Few studies have evaluated this variant, which appears with a relatively high frequency (MAF = 0.199) in our group.

Flavin monooxygenase (FMO) enzymes are catalysts in the oxidative metabolism of many drugs including morphine, tamoxifen, imipramine, and others. The FMO enzymes resemble CYP enzymes in their activity; however, it is a smaller group of enzymes with five members only, which are scarcely studied^57^. In the current study, three variants (rs2266780, rs1736557, and rs2266782) were found. These variants are in *FMO3,* the most important and studied member of *FMO* genes^57^, however, it has never been considered before in any Arab population.

Carboxylesterases (CES) are crucial enzymes in the biotransformation of clinically important drugs such as anticoagulants, antihyperlipidemic, and chemotherapeutics. *CES* genes are known to be highly polymorphic with many SNPs^58^. In the current study, one identified SNP, rs71647871 in *CES1*, has a clinical annotation in PharmGKB supporting its association with clopidogrel efficacy and other associations with lower significance. Rs71647871was detected at MAF = 0.02, which was significantly higher than its MAF in South and East Asians and Africans (Table 4). Despite the importance of *CES1* in metabolizing many compounds, it is relatively understudied compared to *CYP* genes^59^. The comparatively high frequency of this allele in the current group suggests it as a candidate allele for association studies in our population.

N-acetyl transferases (NAT) are key conjugation enzymes that affect multiple pharmacological and environmental compounds. The “acetylator trait” describing differences in drugs’ acetylation was one of the first identified PGx traits^60^. *NAT2* alleles are known to exhibit three phenotypes: rapid, intermediate, and slow acetylators with a variable presentation of these alleles among different populations^61^. Among the few pharmacogenetic studies in the Emirati population, *NAT2* was the most studied pharmacogene^62,63^. In the current group, analysis of *NAT2* alleles and predicted phenotype revealed that more than 81% of our participants were either heterozygous or homozygous for two slow-acetylator alleles (*5,*6 or *7), which was not significantly different (*p* = 0.506) from Al-Ahmed and colleagues observation of 74.4% participants with slow-metabolizer alleles^62^. Some previous studies have pointed to the high frequency of slow metabolizers among different Arab populations^64,65^. The predominance of slow acetylator alleles in Arabs is similar to Europeans and in contrast to East Asian and African populations^66^. Most of the association studies of *NAT2* in Arab countries were focusing on cancer susceptibility^67,68^ rather than its interaction with isoniazid, the antituberculosis drug. There is a strong accumulating evidence supporting *NAT2-*isoniazid interaction and the ultra-slow acetylators susceptibility to isoniazid-induced liver injury^61^. Accordingly, we suggest further evaluation of *NAT2*-antituberculosis drug interaction in Arab populations; nevertheless, tuberculosis is still a notable health problem in some of these countries^69^.

Glutathione transferases (GSTs) are a group of multifunctional proteins that are divided upon their cellular localization into three major families. The cytosolic GSTs are the most studied in human health and disease. Some members of GSTs are known to be polymorphic like *GSTM1*, *GSTT1*, *GSTP1, *and* GSTA1*^70^. In the current study, 14 variants were reported from the four previously mentioned genes. However, only one SNP, rs1695 in *GSTP1*, was of clinical significance (has multiple level-2 and level-3 annotations). Indeed, the most common polymorphism in both *GSTM1* and *GSTT1 *is the complete deletion of the gene, i.e., null allele^70^, which we were not able to detect here. Rs1695, present in the current group at MAF = 0.316, is a substitution that results in decreasing GSTP1 activity. This variant has been studied multiple times as an affecter on the response and outcomes of different tumors with inconsistent findings^71^. The relatively high frequency of the minor allele suggests it as a suitable candidate for population studies.

Dihydropyrimidine dehydrogenase (DPYD) is the rate-limiting enzyme in pyrimidine pathway. It is encoded by *DPYD*, a gene with 23 exons, more than 900 kb, and around 160 variant alleles described in the literature^72^. In PharmGKB, ten different *DPYD *SNPs are annotated with a level-1 association with fluorouracil or/and capecitabin. Different PGx societies have issued fluorouracil and capecitabine prescribing guidelines based on DPYD activity^72^. In the current group, only one of the level-1 actionable *DPYD* variants, rs115232898, was detected in a single heterozygous. Six more variants were detected in the same gene, of which four have a level-3 clinical annotation. These variants are the key SNPs of DPYD*4 (c. 1601G>A), DPYD*5 (c.1627A >G), and DPYD*6 (c.2194G>A), besides rs2297595. Interestingly, the detected alleles were found at similar frequencies in previous studies in Tunisian and Egyptian populations^73,74^. The few numbers of studies covering *DPYD *in Arab populations despite its importance underscores it for further sequencing and association studies.

Thiopurine S-methyltransferase (TPMT) is an enzyme that catalyzes thiopurines, including 6-mercaptopurine (6-MP) and azathioprine metabolism. The association of *TPMT *with thiopurines toxicity, especially 6-MP in pediatric leukemia, has been the role model of PGx implication in the clinic^75,76^. None of the *TPMT* low activity alleles were detected in the current group. Two individuals were heterozygous for TPMT*8 and TPMT*16, which have a moderate significance. TPMT*8 was previously reported from an African American who demonstrated intermediate TPMT activity, while TPMT*16 was reported from one Moroccan and one Caucasian and both showed intermediate TPMT activity^77^. Among Arabs, some studies have evaluated the frequencies of the known star alleles in *TPMT* in healthy or pediatric leukemia participants from Egypt, Tunisia, Morocco, Jordan, and Lebanon^78–82^. The frequencies of the examined low function haplotypes were detected in rates ranging from zero (none of the low functional alleles were found) to 0.0089. However, all these studies used the common *TPMT* star alleles found from studies in Caucasians. The utilization of *TPMT* star alleles to predict phenotype and dose adjustment suffers from the ignorance of rare and novel variants’ impact. One recent study illustrated how the integration of NGS-retrieved common, rare, and novel variants together from both *TPMT* and *NUDT15* is a more reliable predictor of 6-MP dose than the star allele haplotypes^83^.

Nucleoside diphosphate-linked moiety X motif 15 (NUDT15) is believed to de-phosphorylate an active metabolite of thiopurines. GWAS studies have pointed a strong association between *NUDT15* variants and myelosuppression induced by 6-MP. Further studies proved this association and illustrated the inter-population differences in *NUDT15* variations frequencies^84,85^. In the current group, only two variants were detected, of which each was present as a heterozygous allele in one individual (MAF = 0.005). One of these, rs116855232, is the key variant of NUDT15*3, a low functional allele with level-1 clinical annotation in PharmGKB. It is included in the latest CPIC recommendations regarding thiopurine dosing^86^. Since proving its association with 6-MP toxicity, rs116855232 has been repeatedly investigated in Asian and European populations and was present with higher frequencies among Asians^83^. Moreover, it was detected in 2 studies from the middle east with rates of 0.006 and 0.004^82,87^, which was not significantly different (p > 0.05) from its frequency in the current population. Given the importance of *NUDT15* as a predictor of 6-MP dose and the scarce information gathered about its common and rare variants in our geographic area, we recommend further exploration of this gene and applying more association studies.

Vitamin K epoxide reductase (VKORC1) is a crucial enzyme in the vitamin K cycle, and the pharmacological target of warfarin. Several studies have explored variations in *VKORC1 *in independent populations. Our group has previously studied variants in *VKORC1 *in Emiratis^88,89^. The most common SNP in this older group was rs9923231 with AF = 0.52. However, this variant relies upon the 5′ flanking region, and it was not covered in the currently targeted sequencing, which might explain its absence in the present group from the same population. However, one rare variant (rs61742245) reported in Ashkenazi Jewish, Ethiopians, and the former Emirati group was found again in the current group. rs61742245 is associated with the requirement of higher doses of warfarin. Still, it has a very low minor allele frequency worldwide that it can be considered a population-specific variant^89^. Given the earlier described warfarin-associated variants in both *CYP2C9* and *CYP4F2*, we suggest these two genes besides *VKORC1 *as outstanding candidates for further studies in our population, nevertheless, warfarin is still considered the most popular anticoagulant worldwide^90^.

Finally, the interpopulation comparison of MAFs (Table 4) demonstrates the significantly different alleles architecture and genetic profile of the UAE population. Moreover, our heatmap illustrated the clustering of our population close to the South Asia population and far from Africans, which is in concordance with one previous phylogenetic study on 109 Emirati individuals^91^. UAE population also clustered close to the GME populations, which is an expected observation as Emiratis are represented in this variome database. The country's geographical spot, its substructure, and its high consanguinity rates can partially explain these findings, however larger studies are warranted for deeper understanding of pharmacogenomic alleles distribution in Emirati population^91,92^.

There are a few limitations to this study. Although the panel covered a long stretch of the genome (i.e., 159 kbp), it did not include the non-coding regions. Accordingly, the full interpretation of haplotypes and star alleles from some genes was not possible, and the extracted haplotypes might not be definitive in some cases. Besides, some genes like *CYP2D6* is known to present with multiple copies affecting its predicted phenotype. Using further tests for *CYP2D6* and adding some intronic and promotor regions to specific genes is warranted for the optimal haplotyping and phenotype prediction. Finally, we cannot fully represent the UAE admixture population in the current sample, and future studies in the same population should consider larger samples that examine interpopulation stratification.

## Conclusion

In the current analysis, we have illustrated the main candidate pharmacogenes for testing in the UAE population. Furthermore, we have shown how understudied communities might be repertoires of genomic variations, in terms of significantly different allele frequencies and rare and novel variants. Exploring this variation landscape could be utilized for a better understanding of human variation in drug response as well as in health and disease.

Importantly, many of the high frequency variants interact with cardiovascular drugs; like variants in *CYP2C9* and *CYP4F6* interacting with warfarin, *SLCO1B1* with simvastatin and clopidogrel, *CYP2C19* with clopidogrel, and *ABCB1* with simvastatin and digoxin. Cardiovascular diseases are the leading cause of death in UAE^93^; hence PGx studies in cardiovascular drugs are potentially with high significance to the community’s public health.

We also underscored variants interacting with some essential drugs like antiepileptics, antidepressants, anti-infections, and anti-cancer agents. Several detected variants have limited accumulated evidence about their actionability. These variants require further studies to prove, or disapprove, their associations and to move them forward from laboratories into clinics. We highlighted those occurring with high frequencies in our population to encourage further PGx research in our area. We anticipate that applying PGx studies in understudied populations will benefit these specific populations and contribute significantly to the PGx general knowledge.

Our comparisons illustrated consistency with the very few data originating from our region. However, large comparative studies amongst different Arab populations, are immensely needed. Such assessments would likely show significant inter-population differences similar to those reported from comparative studies between Europeans^11^.

Finally, with the growing global interest in preemptive PGx testing, we emphasize the importance of PGx-population studies as an indispensable preparatory step for understudied populations. Herein, we recall the high risk stemming from generalizing findings across populations and from disregarding private variations.

## Supplementary information

Supplementary Information
