# Supplementary material for: Variation in 100 relevant pharmacogenes among emiratis with insights from understudied populations
Source: Sci Rep. 2020 Dec 4;10:21310. doi: 10.1038/s41598-020-78231-3 (PMC7718919; doi:10.1038/s41598-020-78231-3)
Supplement: Supplementary file 1 — Supplementary Information [file 41598_2020_78231_MOESM1_ESM.pdf]

Supplementary Material for:

Variation in 100 Relevant Pharmacogenes among Emiratis with Insights from Understudied Populations

Zeina N. Al-Mahayri[1], George P. Patrinos[1,2,3], Sukanya Wattanapokayakit[4], Nareenart Iemwimangsa[5], Koya Fukunaga[6], Taisei Mushiroda[6], Wasun Chantratita[5], Bassam R. Ali[1,3,7,*]

S1. Pharmacogenes subjected to targeted sequencing.

Gene function	Gene class	Gene family	Number of targeted genes	Genes
Transportation	solute carriers	*SLC*	26	*SLC16A7, SLC19A1, SLC22A1, SLC22A2, SLC22A3, SLC22A4, SLC22A5, SLC22A6, SLC22A8, SLC22A9, SLC22A11, SLC22A12, SLC28A1, SLC28A2, SLC28A3, SLC29A1, SLC29A2, SLC29A3, SLC31A1, SLC46A1, SLC47A1, SLC47A2*
Transportation	solute carriers	*SLCO*	3	*SLCO1B1, SLCO1B3, SLCO2B1*
Transportation	ATP-binding cassette transporters	*ABC*	8	*ABCB1, ABCB4, ABCB11, ABCC1, ABCC2, ABCC3, ABCC4, ABCG2*
Metabolism	Cytochrome P-450 metabolizers	*CYP*	30	*CYP1A1, CYP1A2, CYP1B1, CYP2A6, CYP2A13, CYP2B6, CYP2C8, CYP2C9, CYP2C18, CYP2C19, CYP2D6, CYP2E1, CYP2J2, CYP2S1, CYP2W1, CYP3A4, CYP3A5, CYP3A7, CYP3A43, CYP4A11, CYP4B1, CYP4F2, CYP4F3, CYP4F8, CYP4F12, CYP4Z1, CYP11A1, CYP17A1, CYP19A1, CYP26A1*
Metabolism	Flavin monooxygenase	*FMO*	5	*FMO1, FMO2, FMO3, FMO4, FMO5*
Metabolism	Carboxylases	*CES*	2	*CES1, CES2*
Metabolism	Glutathione S-transferases	*GST*	4	*GSTA1, GSTM1, GSTP1, GSTT1*
Metabolism	N-acetyltransferases	*NAT*	2	*NAT1, NAT2*
Metabolism	Uridine diphosphate glucuronosyltransferase	*UGT*	10	*UGT1A1, UGT1A3, UGT1A4, UGT1A5, UGT1A6, UGT1A7, UGT1A8, UGT1A9, UGT1A10, UGT2B7*
Metabolism	Sulfonyl transferases	*SULT*	4	*SLUT1A1, SULT1A2, SULT1E1, SULT2B1*
Metabolism	Miscellaneous	-	5	*DPYD, TPMT, NUDT1, NUDT15, POR*
Drug target	Reductases	*VKORC*	1	*VKORC1*

S2_Novel variants with CADD phred score >20 (Damaging prediction)

Chr	Start	End	Ref	Alt	Gene.refGene	ExonicFunc.refGene	ExAC_ALL	avsnp147	AF	CADD_raw	CADD_phred
1	47398490	47398490	A	G	CYP4A11	nonsynonymous SNV	.	.	0.005102	5.803	27.1
1	47398686	47398686	T	C	CYP4A11	nonsynonymous SNV	.	.	0.005102	4.753	24.7
1	47533257	47533257	T	C	CYP4Z1	nonsynonymous SNV	.	.	0.005051	4.104	23.7
1	60377419	60377419	A	G	CYP2J2	nonsynonymous SNV	.	.	0.005051	2.783	21.3
1	60377933	60377933	G	C	CYP2J2	nonsynonymous SNV	.	.	0.005051	3.314	22.9
1	97771744	97771744	G	C	DPYD;DPYD-AS1	nonsynonymous SNV	.	.	0.005	5.877	27.4
1	110231722	1.1E+08	T	C	GSTM1	nonsynonymous SNV	.	.	0.007246	4.539	24.3
1	110231855	1.1E+08	A	G	GSTM1	nonsynonymous SNV	.	.	0.007353	3.338	22.9
1	146673046	1.47E+08	T	C	FMO5	nonsynonymous SNV	.	.	0.005051	4.507	24.3
1	171086455	1.71E+08	A	G	FMO3	nonsynonymous SNV	.	.	0.005	4.617	24.5
1	171174446	1.71E+08	G	C	FMO2	unknown	.	rs868719045	0.005051	6.013	27.9
1	171177976	1.71E+08	G	A	FMO2	unknown	.	.	0.005102	3.719	23.3
2	38301891	38301891	T	C	CYP1B1	nonsynonymous SNV	.	.	0.005	5.785	27.1
2	38301919	38301919	T	C	CYP1B1	nonsynonymous SNV	.	.	0.005	3.757	23.3
2	38302003	38302003	G	C	CYP1B1	nonsynonymous SNV	.	.	0.005	3.945	23.6
2	38302203	38302203	T	C	CYP1B1	nonsynonymous SNV	.	.	0.005	3.065	22.4
2	38302485	38302485	A	T	CYP1B1	nonsynonymous SNV	.	.	0.005051	4.75	24.7
2	38302528	38302528	C	T	CYP1B1	nonsynonymous SNV	.	.	0.005051	3.511	23.1
2	169791707	1.7E+08	T	C	ABCB11	nonsynonymous SNV	.	.	0.005051	3.022	22.3
2	169842767	1.7E+08	C	A	ABCB11	nonsynonymous SNV	.	rs770497192	0.005102	5.836	27.2
2	169847360	1.7E+08	A	G	ABCB11	nonsynonymous SNV	.	.	0.005051	6.014	27.9
2	169874586	1.7E+08	T	C	ABCB11	nonsynonymous SNV	.	.	0.005051	3.546	23.1
2	234580951	2.35E+08	G	A	UGT1A9	nonsynonymous SNV	.	.	0.005102	4.676	24.5
2	234581181	2.35E+08	A	G	UGT1A9	nonsynonymous SNV	.	.	0.005102	4.228	23.9
2	234602099	2.35E+08	A	G	UGT1A6	nonsynonymous SNV	.	.	0.005102	6.377	29.5

2	234627986	2.35E+08	A	G	UGT1A4	nonsynonymous SNV	.	.	0.005051	3.096	22.5
4	69962624	69962624	A	T	UGT2B7	nonsynonymous SNV	.	.	0.005102	2.848	21.6
4	69962678	69962678	T	A	UGT2B7	nonsynonymous SNV	.	.	0.005	3.94	23.5
4	69962833	69962833	T	C	UGT2B7	nonsynonymous SNV	.	.	0.005	3.762	23.3
4	69964405	69964405	A	G	UGT2B7	nonsynonymous SNV	.	.	0.005051	4.323	24
4	69978210	69978210	A	G	UGT2B7	nonsynonymous SNV	.	.	0.005	4.573	24.4
4	69978398	69978398	T	C	UGT2B7	nonsynonymous SNV	.	.	0.005	3.233	22.8
4	89016706	89016706	A	G	ABCG2	nonsynonymous SNV	.	.	0.005051	6.543	31
4	89022467	89022467	C	T	ABCG2	nonsynonymous SNV	.	.	0.005102	7.324	34
5	131630421	1.32E+08	T	A	SLC22A4	nonsynonymous SNV	.	.	0.005051	2.903	21.9
5	131705914	1.32E+08	T	C	SLC22A5	nonsynonymous SNV	.	.	0.005	5.27	25.7
5	131726410	1.32E+08	C	T	SLC22A5	nonsynonymous SNV	.	.	0.005051	5.784	27.1
6	18130982	18130982	T	A	TPMT	stopgain	.	.	0.005	9.172	35
6	18139240	18139240	A	G	TPMT	nonsynonymous SNV	.	.	0.005051	6.422	29.7
6	44201213	44201213	T	C	SLC29A1	nonsynonymous SNV	.	.	0.005102	6.866	33
6	160554991	1.61E+08	A	C	SLC22A1	nonsynonymous SNV	.	.	0.005102	5.062	25.2
6	160560780	1.61E+08	A	G	SLC22A1	nonsynonymous SNV	.	.	0.005	5.309	25.8
6	160679386	1.61E+08	A	G	SLC22A2	nonsynonymous SNV	.	.	0.005102	5.995	27.8
7	1024127	1024127	T	C	CYP2W1	nonsynonymous SNV	.	.	0.005051	4.039	23.7
7	1026417	1026417	T	C	CYP2W1	nonsynonymous SNV	.	.	0.005	4.715	24.6
7	2290503	2290503	T	C	NUDT1	nonsynonymous SNV	.	.	0.005051	5.855	27.3
7	75614388	75614388	A	T	POR	nonsynonymous SNV	.	.	0.0102	2.658	20.6
7	75614395	75614395	T	C	POR	nonsynonymous SNV	.	.	0.005102	3.666	23.2
7	87031509	87031509	C	A	ABCB4	nonsynonymous SNV	.	.	0.005102	5.958	27.7
7	87046792	87046792	C	G	ABCB4	nonsynonymous SNV	.	.	0.005051	4.62	24.5
7	87133567	87133567	G	T	ABCB1	nonsynonymous SNV	.	.	0.005155	5.148	25.4
7	99366042	99366042	G	A	CYP3A4	nonsynonymous SNV	.	.	0.0102	3.465	23
7	99366042	99366042	G	C	CYP3A4	nonsynonymous SNV	.	.	0.0102	2.63	20.4

7	99457509	99457509	G	A	CYP3A43	nonsynonymous SNV	.	.	0.005051	6.001	27.8
8	18080182	18080182	T	C	NAT1	nonsynonymous SNV	.	.	0.005	3.157	22.6
8	18257514	18257514	A	G	NAT2	startloss	.	.	0.005051	4.583	24.4
9	86924601	86924601	T	C	SLC28A3	nonsynonymous SNV	.	.	0.005051	3.893	23.5
9	116022598	1.16E+08	A	T	SLC31A1	nonsynonymous SNV	.	.	0.005051	5.205	25.5
9	116022713	1.16E+08	A	G	SLC31A1	nonsynonymous SNV	.	.	0.005051	2.884	21.8
10	73111489	73111489	G	T	SLC29A3	nonsynonymous SNV	.	.	0.005102	6.296	29.1
10	73111540	73111540	T	A	SLC29A3	nonsynonymous SNV	.	.	0.005102	5.624	26.6
10	73122211	73122211	G	T	SLC29A3	nonsynonymous SNV	.	.	0.1313	6.618	32
10	73122213	73122213	A	G	SLC29A3	nonsynonymous SNV	.	.	0.1313	5.691	26.8
10	94834630	94834630	G	T	CYP26A1	nonsynonymous SNV	.	.	0.005051	2.616	20.3
10	94834804	94834804	T	A	CYP26A1	nonsynonymous SNV	.	.	0.005051	6.708	32
10	96535147	96535147	G	A	CYP2C19	nonsynonymous SNV	.	rs267602634	0.005	4.419	24.2
10	96541750	96541750	A	G	CYP2C19	nonsynonymous SNV	.	.	0.005051	2.929	22
10	96602690	96602690	A	T	CYP2C19	nonsynonymous SNV	.	.	0.005	4.339	24
10	96698537	96698537	C	A	CYP2C9	nonsynonymous SNV	.	.	0.005051	5.899	27.5
10	96707610	96707610	C	A	CYP2C9	nonsynonymous SNV	.	.	0.0102	6.74	32
10	96731922	96731922	T	C	CYP2C9	nonsynonymous SNV	.	.	0.005	3.758	23.3
10	96797037	96797037	C	T	CYP2C8	nonsynonymous SNV	.	.	0.005102	6.199	28.7
10	101567852	1.02E+08	A	G	ABCC2	nonsynonymous SNV	.	.	0.005	5.085	25.3
10	101567934	1.02E+08	T	C	ABCC2	nonsynonymous SNV	.	.	0.005	5.545	26.4
10	101605399	1.02E+08	A	G	ABCC2	nonsynonymous SNV	.	.	0.005102	6.604	31
10	104590479	1.05E+08	G	A	CYP17A1	stopgain	.	.	0.005102	12.175	38
10	104590695	1.05E+08	T	A	CYP17A1	nonsynonymous SNV	.	.	0.005051	5.301	25.8
10	135350627	1.35E+08	A	G	CYP2E1	nonsynonymous SNV	.	.	0.005	4.461	24.2
11	62744825	62744825	G	A	SLC22A6	stopgain	.	.	0.005	13.337	41
11	62747209	62747209	G	A	SLC22A6	stopgain	.	.	0.005	8.792	35
11	62748808	62748808	T	C	SLC22A6	nonsynonymous SNV	.	.	0.01515	4.669	24.5

11	62748815	62748815	T	C	SLC22A6	nonsynonymous SNV	.	.	0.0101	5.396	26
11	62763502	62763502	T	A	SLC22A8	nonsynonymous SNV	.	.	0.005051	6.409	29.6
11	64337233	64337233	G	A	SLC22A11	nonsynonymous SNV	.	.	0.005051	3.62	23.2
11	64359177	64359177	G	A	SLC22A12	stopgain	.	.	0.005102	9.488	35
11	66131831	66131831	A	G	SLC29A2	nonsynonymous SNV	.	.	0.005882	7.137	34
11	66133426	66133426	A	G	SLC29A2	nonsynonymous SNV	.	.	0.005051	6.363	29.4
11	74880717	74880717	T	C	SLCO2B1	nonsynonymous SNV	.	.	0.005051	6.094	28.2
12	21327619	21327619	C	T	SLCO1B1	nonsynonymous SNV	.	.	0.005051	6.018	27.9
12	21358845	21358845	T	A	SLCO1B1	nonsynonymous SNV	.	.	0.005	5.306	25.8
13	48615181	48615181	T	C	NUDT15	nonsynonymous SNV	.	rs533746264	0.005102	4.704	24.6
13	95714966	95714966	T	C	ABCC4	nonsynonymous SNV	.	.	0.005102	3.869	23.5
13	95818621	95818621	C	T	ABCC4	nonsynonymous SNV	.	.	0.005102	6.968	33
13	99361860	99361860	A	G	SLC15A1	nonsynonymous SNV	.	.	0.005102	5.481	26.2
15	45556893	45556893	T	C	SLC28A2	nonsynonymous SNV	.	.	0.005102	6.076	28.1
15	75013663	75013663	T	C	CYP1A1	nonsynonymous SNV	.	.	0.005	4.173	23.8
15	75015081	75015081	T	C	CYP1A1	nonsynonymous SNV	.	.	0.005051	3.453	23
15	75015152	75015152	A	G	CYP1A1	nonsynonymous SNV	.	.	0.005	5.487	26.2
15	75015332	75015332	C	A	CYP1A1	nonsynonymous SNV	.	.	0.005051	4.894	24.9
15	75042365	75042365	C	T	CYP1A2	stopgain	.	.	0.005	11.035	37
15	75042413	75042413	T	C	CYP1A2	nonsynonymous SNV	.	.	0.005051	2.855	21.6
15	85438237	85438237	T	C	SLC28A1	nonsynonymous SNV	.	.	0.005	4.193	23.9
15	85447402	85447402	T	C	SLC28A1	nonsynonymous SNV	.	.	0.005051	3.954	23.6
15	85476425	85476425	C	T	SLC28A1	nonsynonymous SNV	.	.	0.005051	7.095	33
16	16108422	16108422	G	A	ABCC1	stopgain	.	.	0.005102	11.588	37
16	16232256	16232256	C	T	ABCC1	nonsynonymous SNV	.	.	0.005	7.632	35
16	16232286	16232286	T	C	ABCC1	nonsynonymous SNV	.	.	0.005	6.323	29.2
16	28618282	28618282	C	T	SULT1A1	nonsynonymous SNV	.	.	0.005102	3.822	23.4
16	31105978	31105978	A	G	VKORC1	nonsynonymous SNV	.	.	0.005	5.418	26

16	55844501	55844501	C	G	CES1	nonsynonymous SNV	.	.	0.005051	3.204	22.7
16	55857516	55857516	T	C	CES1	nonsynonymous SNV	.	.	0.005102	5.158	25.4
16	66974249	66974249	G	A	CES2	nonsynonymous SNV	.	.	0.005102	6.77	32
16	66974252	66974252	T	C	CES2	nonsynonymous SNV	.	.	0.005102	6.195	28.7
16	66977849	66977849	G	C	CES2	nonsynonymous SNV	.	.	0.005051	4.761	24.7
17	19458558	19458558	G	A	SLC47A1	nonsynonymous SNV	.	.	0.005051	4.168	23.8
17	19606429	19606429	A	G	SLC47A2	nonsynonymous SNV	.	.	0.005102	4.622	24.5
17	48746509	48746509	T	C	ABCC3	nonsynonymous SNV	.	.	0.005	3.023	22.3
17	48750474	48750474	G	C	ABCC3	nonsynonymous SNV	.	.	0.005051	5.66	26.7
17	48753309	48753309	T	A	ABCC3	stopgain	.	.	0.005	7.766	35
17	48753316	48753316	T	G	ABCC3	nonsynonymous SNV	.	.	0.005	2.982	22.2
17	48764946	48764946	A	C	ABCC3	nonsynonymous SNV	.	.	0.005051	3.957	23.6
17	48764998	48764998	A	T	ABCC3	nonsynonymous SNV	.	.	0.005051	5.079	25.3
17	48765078	48765078	A	G	ABCC3	nonsynonymous SNV	.	.	0.005051	3.822	23.4
17	48768491	48768491	A	T	ABCC3	nonsynonymous SNV	.	.	0.005051	6.442	29.8
17	48768536	48768536	T	C	ABCC3	nonsynonymous SNV	.	.	0.005051	5.971	27.7
19	15752376	15752376	T	C	CYP4F3	nonsynonymous SNV	.	.	0.005	4.24	23.9
19	15763647	15763647	G	A	CYP4F3	nonsynonymous SNV	.	.	0.005051	5.148	25.4
19	15769558	15769558	G	A	CYP4F3	nonsynonymous SNV	.	.	0.005	2.64	20.4
19	15770170	15770170	G	A	CYP4F3	stopgain	.	.	0.005102	10.841	36
19	15807870	15807870	A	G	CYP4F12	nonsynonymous SNV	.	.	0.005	4.883	24.9
19	15989588	15989588	A	G	CYP4F2	nonsynonymous SNV	.	.	0.005	5.225	25.6
19	15990421	15990421	T	C	CYP4F2	nonsynonymous SNV	.	.	0.005051	4.771	24.7
19	41510017	41510017	T	C	CYP2B6	nonsynonymous SNV	.	.	0.005	4.84	24.8
19	41601700	41601700	A	G	CYP2A13	nonsynonymous SNV	.	.	0.005	5.368	25.9
19	41601732	41601732	G	A	CYP2A13	nonsynonymous SNV	.	.	0.005	3.938	23.5
21	46945819	46945819	A	G	SLC19A1	nonsynonymous SNV	.	.	0.005051	3.61	23.2
21	46957732	46957732	T	C	SLC19A1	nonsynonymous SNV	.	.	0.01333	2.864	21.7

21	46957774	46957774	A	G	SLC19A1	nonsynonymous SNV	.	.	0.01765	2.917	21.9
22	42523948	42523948	T	A	CYP2D6	nonsynonymous SNV	.	.	0.0101	4.604	24.4
22	42524180	42524180	T	C	CYP2D6	nonsynonymous SNV	.	.	0.005051	4.073	23.7
22	42525793	42525793	T	G	CYP2D6	nonsynonymous SNV	.	.	0.0101	4.273	24

S3-a. Star alleles, their associated SNPs and frequencies

Gene	Detected Star Alelle	Associated SNP	Frequency	No. of Heterozygous	No. of Homozygous
**CYP1A1**	*2C	rs1048943	0.041	8	0
**CYP1A1**	*4	rs1799814	0.189	25	6
**CYP1B1**	*2	rs10012	0.354	42	14
**CYP1B1**	*2	rs1056827	0.355	43	14
**CYP1B1**	*3	rs1056836	0.641	37	45
**CYP1B1**	*4	rs1800440	0.168	23	5
**CYP1B1**	*6	*2 +*3 variants			
**CYP1B1**	*12	rs28936700	0.01	2	0
**CYP2A6**	*2	rs1801272	0.025	5	0
**CYP2A6**	*18	rs1809810	0.995	0	97
**CYP2B6**	*2	rs8192709	0.116	21	1
**CYP2B6**	*3	rs45482602	0.026	1	2
**CYP2B6**	*4	rs2279343	0.059	4	3
**CYP2B6**	*5	rs3211371	0.07	12	1
**CYP2B6**	*6	*4 + *9 variants		28	6
**CYP2B6**	*9	rs3745274	0.31	7	8
**CYP2B6**	*11	rs35303484	0.015	3	0
**CYP2B6**	*12	rs36060847	0.005	1	0
**CYP2B6**	*18	rs28399499	0.01	2	0
**CYP2A13**	*2	rs8192784	0.055	11	0
**CYP2A13**	*2	rs8192789	0.046	9	0
**CYP2A13**	*7	rs72552266	0.005	1	0
**CYP2A13**	*8	rs112337232	0.01	2	0
**CYP2C8**	*2	rs11572103	0.03	6	0
**CYP2C8**	*3	rs11572080	0.141	24	2
**CYP2C8**	*3	rs10509681	0.136	23	2

**CYP2C8**	*4	rs1058930	0.015	3	0
**CYP2C9**	*2	rs1799853	0.11	18	2
**CYP2C9**	*3	rs1057910	0.071	14	0
**CYP2C9**	*8	rs7900194	0.02	2	1
**CYP2C9**	*9	rs2256871	0.01	2	0
**CYP2D6**	*2	rs1135840	0.405	49	16
**CYP2D6**	*10	rs1065852	0.153	26	2
**CYP2D6**	*17	rs28371706	0.04	8	0
**CYP2D6**	*29	rs59421388	0.005	1	0
**CYP2D6**	*29	rs61736512	0.005	1	0
**CYP2D6**	*33	rs28371717	0.005	1	0
**CYP2D6**	*35	rs769258	0.015	3	0
**CYP2D6**	*39	rs1135840	0.405	49	16
**CYP2D6**	*39	rs1058164	0.404	46	17
**CYP2D6**	*41	rs16947	0.567	52	30
**CYP2D6**	*41	rs28371725	0.155	21	5
**CYP2D6**	*41	rs1135840	0.405	49	16
**CYP2D6**	*43	rs28371696	0.005	1	0
**CYP2D6**	*112	rs568495591	0.005	1	0
**CYP2D6**	*116	rs141009491	0.015	1	1
**CYP2J2**	*9	rs148429756	0.01	2	0
**CYP2S1**	*3	rs34971233	0.026	5	0
**CYP2W1**	*2	rs3735684	0.055	9	1

**CYP3A4**	*12	rs12721629	0.005	1	0
**CYP3A7**	*2	rs2257401	0.827	28	67
**CYP3A43**	*1B	rs17342647	0.005		
**CYP3A43**	*2A	rs61469810	0.121	20	2
**CYP3A44**	*2B	rs61469810	0.121	20	2
**CYP3A45**	*2B	rs680055	0.136	19	4
**CYP3A46**	*3	rs680055	0.136	19	4
**CYP4B1**	*2	rs3215983	0.102	16	2
**CYP4B1**	*2	rs4646491	0.102	16	2
**CYP4B1**	*2	rs2297810	0.184	22	7
**CYP4B1**	*3	rs4646487	0.155	25	3
**CYP4B1**	*4	rs45467195	0.005	1	0
**CYP4B1**	*5	rs2297810	0.184	22	7
**CYP4B1**	*S1	rs2297809	0.105	15	3
**CYP4F2**	*2	rs3093105	0.2194	37	3
**CYP4F2**	*3	rs2108622	0.4592	54	18
**CYP19A1**	*3	rs28757184	0.015	3	0
**CYP19A1**	*4	rs700519	0.036	5	1
**CYP26A1**	*2	rs61735552	0.05	1	0

**DPYD**	*4	rs1801158	0.04	8	0
**DPYD**	*5	rs1801159	0.157	25	3
**DPYD**	*6	rs1801160	0.165	31	1
**DPYD**	*9A	rs1801265	0.75	35	56
**DPYD**	*S3	rs2297595	0.155	25	3
**DPYD**	*S6	rs115232898	0.005	1	0
**DPYD**	*S21	rs45589337	0.005	1	0
**GSTM1**	*3	rs74837985	0.175	4	9
**GSTP1**	*2	67352689:A>G	0.316	42	10
**GSTP1**	*3	67352689:A>G	0.316	42	10
**GSTP1**	*3	rs1138272	0.111	20	1
**GSTP1**	*4	rs1138272	0.111	20	1
**NAT2**	*5	rs1801280	0.46	53	19
**NAT2**	*6	rs1799930	0.315	47	8
**NAT2**	*7	rs1799931	0.036	7	0
**NAT2**	*11	rs1799929	0.415	55	14
**NAT2**	*12	rs1208	0.526	53	25
**NAT2**	*13	rs1041983	0.33	50	8
**NUDT15**	*3	rs116855232	0.005	1	0
**POR**	*28	rs1057868	0.25	42	4
**SLC15A2**	*2	rs2257212	0.333	50	8

**SLC22A2**	*2	rs624249	0.48	52	21
**SLC22A2**	*3	rs316003	0.833	23	71
**SLC22A2**	*6	rs316019	0.913	13	83
**SLC22A2**	*K432Q	rs8177517	0.02	4	0
**SLCO1B1**	*1B	rs2306283	0.48	55	20
**SLCO1B1**	*5	rs4149056	0.187	31	3
**SLCO1B1**	*14	rs11045819	0.045	9	0
**SLCO1B1**	*15	*1B + *5 variants			
**SLCO1B1**	*35	rs34671512	0.05	10	0
**SLCO1B1**	*4S	rs71581941	0.03	6	0
**SLCO1B3**	*S1	rs7311358	0.835	29	69
**SLCO2B1**	* S464F	rs2306168	0.036	5	1
**SULT1A1**	*2	rs1042028	0.199	21	9
**SULT1A1**	*3	rs1801030	0.964	7	91
**TPMT**	*8	rs56161402	0.005	1	0
**TPMT**	*16	rs144041067	0.005	1	0
**UGT1A1**	*6	rs4148323	0.005	1	0
**UGT1A1**	*28	rs8175347 (7 TA repeats)	0.645	13	58
**UGT1A1**	*37	rs8175347 (8 TA repeats)	0.085	7	5
**UGT1A4**	*2	rs6755571	0.045	9	0

**UGT1A4**	*3B	rs2011425	0.117	21	1
**UGT1A4**	*4	rs3892221	0.005	1	0
**UGT2B7**	*2	rs7439366	0.465	46	23

S3-b. Diplotypes, their frequencies and their PharmGKB annotations

Gene	Diplotype	Frequency	PharmGKB annotation (Level of evidence)
**CYP1A1**	*1/*1	0.63	-
**CYP1A1**	*1/*2C	0.06	Breast cancer patients may have increased progression-free survival time when treated with capecitabine and docetaxel (3)
**CYP1A1**	*2C/*4	0.02	-
**CYP1A1**	*4/*4	0.06	-
**CYP1B1**	*1/*1	0.06	
**CYP1B1**	*1/*2	0.09	-
**CYP1B1**	*1/*3	0.08	-
**CYP1B1**	*1/*4	0.07	-
**CYP1B1**	*1/*6	0.06	-
**CYP1B1**	*2/*2	0.11	-
**CYP1B1**	*2/*3	0.13	-
**CYP1B1**	*2/*4	0.09	-
**CYP1B1**	*2/*6	0.03	-
**CYP1B1**	*3/*3	0.14	-
**CYP1B1**	*3/*4	0.05	-
**CYP1B1**	*3/*6	0.03	-
**CYP1B1**	*3/*12	0.01	-

**CYP1B1**	*4/*4	0.04	-
**CYP1B1**	*4/*6	0.01	-
**CYP2A6**	*1/*1	0.02	
**CYP2A6**	*1/*18	0.01	
**CYP2A6**	*2/*18	0.05	May have decreased metabolism of nicotine (2A)
**CYP2A6**	*18/*18	0.92	
**CYP2A13**	*1/*1	0.87	
**CYP2A13**	*1/*2	0.1	-
**CYP2A13**	*1/*7	0.01	-
**CYP2A13**	*1/*8	0.02	-
**CYP2B6**	*1/*1	0.19	
**CYP2B6**	*1/*2	0.16	
**CYP2B6**	*1/*3	0.01	
**CYP2B6**	*1/*5	0.07	
**CYP2B6**	*1/*9	0.25	
**CYP2B6**	*1/*11	0.02	
**CYP2B6**	*1/*12	0.01	
**CYP2B6**	*1/*18	0.02	May have a decreased concentration of hydroxybupropion (2A)
**CYP2B6**	*2/*2	0.01	
**CYP2B6**	*2/*9	0.05	
**CYP2B6**	*3/*9	0.02	
**CYP2B6**	*4/*6	0.01	May have a increased metabolism of bupropion and an increased concentration of hydroxybupropion (2A)
**CYP2B6**	*5/*9	0.06	
**CYP2B6**	*6/*6	0.03	May have decreased metabolism of efavirenz (1A)

**CYP2B6**	*6/*9	0.03	
**CYP2B6**	9/*9	0.05	
**CYP2B6**	*9/*11	0.01	
**CYP2C8**	*1/*1	0.67	-
**CYP2C8**	*1/*2	0.05	-
**CYP2C8**	*1/*3	0.22	-
**CYP2C8**	*1/*4	0.03	May have increased plasma concentration of montelukast (3)
**CYP2C8**	*2/*3	0.01	May have decreased pioglitazone metabolism (3) or decreased plasma concentration (3)
**CYP2C8**	*3/*3	0.02	May have decreased metabolism of ibuprofen (2A)<br>May require decreased dose of ibuprofen (3)<br>Kidney transplantation treated with tacrolimus may have an increased risk of kidney disfunction (3)<br>May have decreased plasma concentration of montelukast and pioglitazone (3)
**CYP2C9**	*1/*1	0.62	
**CYP2C9**	*1/*2	0.17	May decrease metabolism and increase plasma concentration of phenytoin, and increase adverse drug reactions (1A), may Increase risk of over-anticoagulation with warfarin and bleeding and may require a decreased dose (1A), may decrease the metabolism of piroxicam, meloxicam and tenoxicam (1A), may affect the metabolism and exposure to flurbiprofen (1A), may decrease the metabolism and clearance of ibuprofen (1A), may require an altered dose of acenocoumarol and an altered likelihood of over-coagulation (2A).
**CYP2C9**	*1/*3	0.13	May decrease metabolism and increase plasma concentration of phenytoin, and increase adverse drug reactions (1A), may Increase risk of over-anticoagulation with warfarin and bleeding and may require a decreased dose (1A), may decrease the metabolism of piroxicam, meloxicam and tenoxicam (1A), may affect the metabolism and exposure to flurbiprofen (1A), may decrease the metabolism and clearance of ibuprofen (1A), may require an altered dose of acenocoumarol and an altered likelihood of over-coagulation (2A).
**CYP2C9**	*1/*8	0.02	May affect the metabolism of diclofenac and losartan (3)
**CYP2C9**	*1/*9	0.02	-

**CYP2C9**	*2/*2	0.02	May decrease metabolism and increase plasma concentration of phenytoin, and increase adverse drug reactions (1A), may Increase risk of over-anticoagulation with warfarin and bleeding and may require a decreased dose (1A), may decrease the metabolism of meloxicam (1A), may decrease the metabolism and clearance of ibuprofen (1A), may require an altered dose of acenocuomarol and an altered likelihood of over-coagulation (2A).
**CYP2C9**	*2/*3	0.01	May decrease the metabolism and increase plasma concentration of phenytoin, and increase the adverse drug reactions (1A), may Increase risk of over-anticoagulation with warfarin and bleeding and may require a decreased dose (1A), may decrease the metabolism and clearance of ibuprofen (1A), may drastically decrease the metabolism of tenoxicam (1A), may require an altered dose of acenocuomarol and an altered likelihood of over-coagulation (2A).
**CYP2C9**	*8/*8	0.01	-
**CYP2D6***	*1/*1	0.13	-
**CYP2D6**	*1/*2	0.33	-
**CYP2D6**	*1/*10	0.1	May have increased plasma concentration and decreased clearance of paroxetine (1A), May have increased steady state plasm concentration of fluvoxamine (1A)
**CYP2D6**	*1/*17	0.02	
**CYP2D6**	*1/*29	0.01	
**CYP2D6**	*1/*33	0.01	
**CYP2D6**	*1/*35	0.01	
**CYP2D6**	*1/*43	0.01	
**CYP2D6**	*1/*116	0.01	
**CYP2D6**	*2/*2	0.12	
**CYP2D6**	*2/*10	0.09	May have increased plasma concentration and decreased clearance of paroxetine (1A)
**CYP2D6**	*2/*17	0.01	
**CYP2D6**	*2/*35	0.01	
**CYP2D6**	*2/*112	0.01	
**CYP2D6**	*10/*10	0.02	May have increased plasma concentration and decreased clearance of paroxetine (1A),

**CYP2D6**	*41/*41	0.14	may have a decreased metabolism of tamoxifen to its active metabolite endoxifen, and increased likelihood of recurrence and decreased event-free and recurrence-free survival in breast cancer patients (1A)<br>May have decreased metabolism of nortriptyline and increased side effects (1A),<br>May have increased steady state plasma concentration of fluvoxamine and increased GI side effects (1A),<br>May have decreased metabolism or clearance of codeine and decreased response to it (1A),<br>May have decreased clearance of atomoxetine (1A),<br>When treated with amitriptyline may have increased nortriptyline plasma level (1A)<br>May have decreased metabolism of tramadol and should avoid its use (1A),<br>May have decreased metabolism/clearance of venlafaxine and may have decreased tolerance to it (2A),<br>May have increased concentration of tolterodine and its active metabolite (2A),<br>May have decreased metabolism/clearance of metoprolol (2A).<br>May have lower clearance of flecainide (2A),<br>May have decreased metabolism of propafenone and increased side effects (2A)<br>May have reduced metabolism of desipramine (2A),<br>May have decreased metabolism/clearance of risperidone (2A)
**CYP2D6**	*10/*17	0.04	may have a decreased metabolism of tamoxifen to its active metabolite endoxifen, and increased likelihood of recurrence and decreased event-free and recurrence-free survival in breast cancer patients (1A),<br>May have decreased metabolism/clearance of metoprolol (2A).
**CYP2D6**	*10/*35	0.01	
**CYP2D6**	*10/*39	0.02	
**CYP2D6**	*39/*39	0.01	
**CYP2D6**	*45/*45	0.01	May have decreased metabolism/clearance of metoprolol (2A)
**CYP2D6**	*116/*116	0.01	
**CYP2J2**	*1/*1	0.98	-
**CYP2J2**	*1/*9	0.02	-
**CYP2S1**	*1/*1	0.95	-
**CYP2S1**	*1/*3	0.05	-
**CYP2W1**	*1/*1	0.9	-
**CYP2W1**	*1/*2	0.09	-
**CYP2W1**	*2/*2	0.01	-
**CYP3A4**	*1/*1	0.99	-
**CYP3A4**	*1/*12	0.01	May have decreased metabolism of paclitaxel (3)
**CYP3A7**	*1/*1	0.67	-

**CYP3A7**	*1/*2	0.28	-
**CYP3A8**	*2/*2	0.05	-
**CYP3A43**	*1/*1	0.71	
**CYP3A43**	*1/*1B	0.01	-
**CYP3A43**	*1/*2A	0.05	-
**CYP3A43**	*1/*2B	0.14	-
**CYP3A43**	*1/*3	0.05	-
**CYP4B1**	*1/*1	0.45	-
**CYP4B1**	*1/*2	0.09	-
**CYP4B1**	*1/*3	0.21	-
**CYP4B1**	*1/*4	0.01	-
**CYP4B1**	*1/*5	0.08	-
**CYP4B1**	*1/*S1	0.01	-
**CYP4B1**	*2/*2	0.02	-
**CYP4B1**	*2/*3	0.01	-
**CYP4B1**	*2/*5	0.03	-
**CYP4B1**	*3/*3	0.03	-
**CYP4B1**	*3/*5	0.02	-
**CYP4B1**	*5/*5	0.01	-
**CYP4B1**	*S1/*2	0.03	-
**CYP4F2**	*1/*1	0.21	-
**CYP4F2**	*1/*2	0.07	-
**CYP4F2**	*1/*3	0.54	May require a higher dose of warfarin (1A) and acenocoumarol (2A) and phenprocoumon (2A), May have decreased INR when treated with warfarin (3), May have increased concentration, exposure and steady-state levels of vitamin E when taking supplements (3)
**CYP4F2**	*3/*3	0.18	May require a higher dose of warfarin (1A) and acenocoumarol (2A) and phenprocoumon (2A), May have decreased INR when treated with warfarin (3), May have increased concentration, exposure

			and steady-state levels of vitamin E when taking supplements (3),<br>may have increased response to aspirin and clopidogrel in acute coronary syndrome (3)
**CYP19A1**	*1/*1	0.91	-
**CYP19A2**	*1/*3	0.03	-
**CYP19A3**	*1/*4	0.05	-
**CYP19A4**	*4/*4	0.01	-
**CYP26A1**	*1/*1	0.99	-
**DPYD**	*1/*1	0.24	-
**DPYD**	*1/*4	0.03	-
**DPYD**	*1/*5	0.11	-
**DPYD**	*1/*6	0.1	-
**DPYD**	*1/*9A	0.13	-
**DPYD**	*1/*S3	0.03	-
**DPYD**	*4/*6	0.01	-
**DPYD**	*4/*9A	0.03	-
**DPYD**	*4/*S21	0.01	-
**DPYD**	*5/*5	0.03	-
**DPYD**	*5/*6	0.08	-
**DPYD**	*5/*9A	0.04	-
**DPYD**	*5/*S6	0.01	may have an increased risk for flurouracil toxicity (1A)
**DPYD**	*6/*9A	0.07	-
**DPYD**	*9A/*9A	0.02	-
**DPYD**	*S3/*5	0.01	-
**DPYD**	*S3/*6	0.03	-
**DPYD**	*S3/*9A	0.02	-
**GSTM1**	*1/*1	0.86	-
**GSTM1**	*1/*3	0.04	-

**GSTM1**	*3/*3	0.1	-
**GSTP1**	*1/*1	0.45	-
**GSTP1**	*1/*2	0.26	-
**GSTP1**	*1/*3	0.13	-
**GSTP1**	*2/*2	0.05	-
**GSTP1**	*2/*3	0.04	-
**GSTP1**	*2/*4	0.03	-
**GSTP1**	*3/*3	0.01	-
**NAT2**	*4/*4	0.03	-
**NAT2**	*4/*5	0.09	-
**NAT2**	*4/*6	0.05	-
**NAT2**	*4/*12	0.01	-
**NAT2**	*5/*5	0.19	May have a decreased metabolism of isoniazid (2A), may have an increased risk of developing isoniazid-induced hepatotoxicity (2A), may have decreased metabolism of hydralazine (2A)-
**NAT2**	*5/*6	0.32	
**NAT2**	*5/*7	0.04	
**NAT2**	*6/*7	0.01	
**NAT2**	*5/*11	0.16	-
**NAT2**	*5/*12	0.06	-
**NAT2**	*5/*13	0.02	-
**NAT2**	*6/*13	0.08	-
**NAT2**	*7/*11	0.01	-
**NAT2**	*12/*12	0.01	-
**NAT2**	*5/*5	0.19	May have decreased acetylation of isoniazid (2A),<br>may have an increased risk of developing isoniazid induced hepatotoxicity (2A),<br>may have decreased metabolism of hydralazine (2A)<br>and can have an altered response to it (3),<br>may have decreased metabolism of dipyrone (3).
**NAT2**	*6/*6	0.08	May have decreased metabolism of isoniazid (2A),<br>may have an increased risk of developing isoniazid induced hepatotoxicity (2A),<br>may have decreased metabolism of hydralazine (2A)<br>and can have an altered response to it (3),

			may have decreased metabolism of dipyrone (3).
**NAT2**	*6/*7	0.01	May have decreased metabolism of isoniazid (2A),<br>may have an increased risk of developing isoniazid induced hepatotoxicity (2A),<br>may have decreased metabolism of hydralazine (2A)<br>and can have an altered response to it (3),<br>may have decreased metabolism of dipyrone (3).
**NAT2**	*5/*6	0.18	May have decreased metabolism of isoniazid (2A),<br>may have an increased risk of developing isoniazid induced hepatotoxicity (2A),<br>may have decreased metabolism of hydralazine (2A)<br>and can have an altered response to it (3),<br>may have decreased metabolism of dipyrone (3).
**NAT2**	*5/*7	0.02	May have decreased metabolism of isoniazid (2A),<br>may have an increased risk of developing isoniazid induced hepatotoxicity (2A),<br>may have decreased metabolism of hydralazine (2A)<br>and can have an altered response to it (3),<br>may have decreased metabolism of dipyrone (3).
**NUDT15**	*1/*1	0.995	-
**NUDT15**	*1/*3	0.005	May have an increased risk of developing leukopenia, neutropenia or alopecia (1A),<br>may toelarte lower doses of mercaptopurine (2B),<br>May have increased risk of thiopurine induced cytopenia (3)
**POR**	*1/*1	0.54	-
**POR**	*1/*28	0.42	May have decreased trough concentrations of cyclosporine, sirolimus (3),<br>may have changes in metabolism of Midazolam, nicotine (3),<br>may alter sunitinib response in GIST (3),<br>may alter atorvastatin response in familial hypercholesterolemia (3),<br>may alter patient's response to tacrolimus following kidney transplantation (3).
**POR**	*28/*28	0.04	May have decreased trough concentrations of cyclosporine, sirolimus (3),<br>may have changes in metabolism of Midazolam, nicotine (3),<br>may alter sunitinib response in GIST (3),<br>may alter atorvastatin response in familial hypercholesterolemia (3),<br>may alter patient's response to tacrolimus following kidney transplantation (3).
**SLC15A2**	*1/*1	0.43	-
**SLC15A2**	*1/*2	0.49	May affect response to sorafenib in hepatocellular carcinoma patients (3)
**SLC15A2**	*2/*2	0.08	May affect response to sorafenib in hepatocellular carcinoma patients (3)
**SLC22A2**	*1/*1	0.12	-
**SLC22A2**	*1/*2	0.38	-

**SLC22A2**	*1/*3	0.08	-
**SLC22A2**	*1/*6	0.01	May have altered clearance of L-tryptophan and metformin (3),<br>may have altered risk of toxicity and ototoxicity with cisplatin (3),<br>may have increased risk of anthracycline induced cardiotoxicity in children with neoplasms (3),<br>may have altered risk of platinum compounds induced hepatotoxicity (3)
**SLC22A2**	*1/*K432Q	0.01	-
**SLC22A2**	*2/*2	0.21	-
**SLC22A2**	*2/*3	0.12	-
**SLC22A2**	*2/*6	0.01	May have altered clearance of L-tryptophan and metformin (3),<br>may have altered risk of toxicity and ototoxicity with cisplatin (3),<br>may have increased risk of anthracycline induced cardiotoxicity in children with neoplasms (3),<br>may have altered risk of platinum compounds induced hepatotoxicity (3)
**SLC22A2**	*2/*K432Q	0.01	-
**SLC22A2**	*3/*3	0.03	-
**SLC22A2**	*3/*K432Q	0.01	-
**SLC22A2**	*6/*6	0.01	May have altered clearance of L-tryptophan and metformin (3),<br>may have altered risk of toxicity and ototoxicity with cisplatin (3),<br>may have increased risk of anthracycline induced cardiotoxicity in children with neoplasms (3),<br>may have altered risk of platinum compounds induced hepatotoxicity (3)
**SLCO1B1**	*1/*1	0.23	-
**SLCO1B1**	*1/*1B	0.2	-
**SLCO1B1**	*1/*5	0.02	-
**SLCO1B1**	*1/*14	0.05	-
**SLCO1B1**	*1/*15	0.14	May have increased exposure to pitvastatin (3)
**SLCO1B1**	*1/*35	0.07	-
**SLCO1B1**	*1/*4S	0.04	-
**SLCO1B1**	*1B/*1B	0.07	May have decreased bioavailability of pravastatin (2A),<br>May have decreased concentration of atorvastatin (3)<br>May have decreased exposure to rosuvastatin (3)
**SLCO1B1**	*1B/*5	0.01	-
**SLCO1B1**	*1B/*14	0.02	-
**SLCO1B1**	*1B/*15	0.05	May have decreased concentration of atorvastatin (3)

			May have decreased exposure to rosuvastatin (3)
**SLCO1B1**	*1B/*35	0.05	-
**SLCO1B1**	*1B/*S4	0.01	-
**SLCO1B1**	*5/*15	0.02	-
**SLCO1B1**	*14/*15	0.02	-
**SLCO1B1**	*15/*15	0.01	May have increased bioavailability of pravastatin (2A),<br>May have increased concentration of atorvastatin (3),<br>May have increased exposure to pitvastatin (3),<br>May have increased exposure to rosuvastatin and decreased hepatic intake (3)
**SLCO1B1**	*15/*22	0.01	-
**SLCO1B1**	*S4/*15	0.01	-
**SLCO1B3**	*1/*1	0.02	-
**SLCO1B3**	*1/*S1	0.29	May affect response to mycophenolic acid<br>and mycophenolate mofetil in transplantation patients (3),<br>May affect survival rates and risk of allograft dysfunction in transplant patients<br>treated with mycophenolic acid (3), May have altered clearance of docetaxel (3),<br>may have decreased risk of anemia induced by carboplatin and paclitaxel used in carcinomas (3)
**SLCO1B3**	*S1/*S1	0.69	May affect response to mycophenolic acid<br>and mycophenolate mofetil in transplantation patients (3),<br>May affect survival rates and risk of allograft dysfunction in transplant patients<br>treated with mycophenolic acid (3),<br>May have altered clearance of docetaxel (3),<br>may have decreased risk of anemia induced by carboplatin and paclitaxel used in carcinomas (3)
**SLCO2B1**	*1/*1	0.94	-
**SLCO2B1**	*1/*S464F	0.05	May have decreased area under the plasma concentration-time curve in fexofenadine (3)
**SLCO2B1**	*S464F/*S464F	0.01	May have decreased area under the plasma concentration-time curve in fexofenadine (3)
**SULT1A1**	*1/*1	0.62	-
**SULT1A1**	*1/*2	0.2	-
**SULT1A1**	*1/*3	0.06	-
**SULT1A1**	*2/*2	0.09	-
**SULT1A1**	*2/*3	0.01	-

**SULT1A1**	*3/*3	0.02	-
**TPMT**	*1/*1	0.98	-
**TPMT**	*1/*8	0.01	-
**TPMT**	*1/*16	0.01	-
**UGT1A1**	*1/*1	0.99	-
**UGT1A1**	*1/*6	0.01	may have decreased risk of irinotecan induced neutropenia (2A)<br>may have increased metabolism of SN-38 when treated with irinotecan (2A)<br>May have increased AUC of letermovir (3)<br>may have altered risk of hyperbilirubinemia with indinavir (3),<br>May have increased glucuronidation of carvedilol (3)
**UGT1A4**	*1/*1	0.68	-
**UGT1A4**	*1/*2	0.08	-
**UGT1A4**	*1/*3B	0.21	May have an altered serum concentrations of lamotregine (2B)
**UGT1A4**	*2/*3B	0.01	-
**UGT1A4**	*3B/*3B	0.01	May have an increased serum concentrations of lamotregine and improved response (2B)
**UGT1A4**	*3B/*4	0.01	-
**UGT2B7**	*1/*2	0.46	May decrease the analgesic response to morphine (3),<br>may affect response to oxycodone (3)<br>may affect plasma concentration of methadone<br>and the severity of opiate withdrawal symptoms (3),<br>may affect response to oxcarbazepine (3),<br>may affect plasma concentration of efavirenz (3),<br>may affect response to fentanyl (3)
**UGT2B7**	*2/*2	0.3	May decrease the analgesic response to morphine (3),<br>may affect response to oxycodone (3)<br>may affect plasma concentration of methadone<br>and the severity of opiate withdrawal symptoms (3),<br>may affect response to oxcarbazepine (3),<br>may affect plasma concentration of efavirenz (3),<br>may affect response to fentanyl (3),<br>may require altered dose of codeine and morphine (3)
